# Interannual Dynamics of Ice Cliff Populations on Debris‐Covered Glaciers From Remote Sensing Observations and Stochastic Modeling

**DOI:** 10.1029/2021JF006179

**Published:** 2021-10-13

**Authors:** M. Kneib, E. S. Miles, P. Buri, P. Molnar, M. McCarthy, S. Fugger, F. Pellicciotti

**Affiliations:** ^1^ High Mountain Glaciers and Hydrology (HIMAL) Swiss Federal Institute for Forest Snow and Landscape Research WSL Birmensdorf Switzerland; ^2^ Institute of Environmental Engineering ETH Zurich Zurich Switzerland; ^3^ Department of Geography Northumbria University Newcastle UK

**Keywords:** debris‐covered glaciers, supraglacial ice cliffs, supraglacial ponds, birth‐death model, stochastic processes

## Abstract

Ice cliffs are common on debris‐covered glaciers and have relatively high melt rates due to their direct exposure to incoming radiation. Previous studies have shown that their number and relative area can change considerably from year to year, but this variability has not been explored, in part because available cliff observations are irregular. Here, we systematically mapped and tracked ice cliffs across four debris‐covered glaciers in High Mountain Asia for every late ablation season from 2009 to 2019 using high‐resolution multi‐spectral satellite imagery. We then quantified the processes occurring at the feature scale to train a stochastic birth‐death model to represent the cliff population dynamics. Our results show that while the cliff relative area can change by up to 20% from year to year, the natural long‐term variability is constrained, thus defining a glacier‐specific cliff carrying capacity. In a subsequent step, the inclusion of external drivers related to climate, glacier dynamics, and hydrology highlights the influence of these variables on the cliff population dynamics, which is usually not a direct one due to the complexity and interdependence of the processes taking place at the glacier surface. In some extreme cases (here, a glacier surge), these external drivers may lead to a reorganization of the cliffs at the glacier surface and a change in the natural variability. These results have implications for the melt of debris‐covered glaciers, in addition to showing the high rate of changes at their surface and highlighting some of the links between cliff population and glacier state.

## Key Points

- The proportion of ice cliffs on debris‐covered glaciers can change by up to 20% from 1 year to the next
- There is no direct influence of external drivers on cliff variability due to the complexity and interdependence of glacier surface processes
- The long‐term variability of the cliff population is constrained unless it is modified by an extreme climatic and/or glacier change

## 1 Introduction

Debris‐covered glaciers are widespread in all mountain ranges around the globe (Herreid & Pellicciotti, 2020b; Scherler et al., 2018) and especially in High Mountain Asia (HMA), where half of the glaciers larger than 2 km^2^ have more than 5% of their total area covered by a layer of rock debris (Herreid & Pellicciotti, 2020b) varying in thickness from centimeter to meter scale. These glaciers are often characterized by undulating, hummocky topography (Bartlett et al., 2020) and their surface is punctuated by supraglacial ponds, streams, and ice cliffs. Ice cliffs have been observed in all the main mountain ranges of the planet (Anderson et al., 2021; Benn et al., 2001; Chinn & Dillon, 1987; Herreid & Pellicciotti, 2018; Inoue & Yoshida, 1980; Johnson, 1992; Mölg et al., 2019; Moore, 2018; Ogilvie, 1904; Reid & Brock, 2014; Röhl, 2006; Sakai et al., 1998; Shahgedanova et al., 2005) and have been observed to account for 1%–12% of the total debris‐covered area (Anderson et al., 2021; Brun et al., 2018; Kneib et al., 2020; Reid & Brock, 2014; Sakai et al., 1998). They consist of steep, bare, or very thinly debris‐covered ice faces within the debris‐covered part of the glacier and are often associated with supraglacial streams or ponds (Mölg et al., 2019; Steiner et al., 2019). Cliffs appear when the surface slope is too steep for the debris to remain on it (Moore, 2018). Therefore, ice cliff formation has been suggested to be triggered by several possible mechanisms, including the collapse of englacial conduits (Benn et al., 2012; Immerzeel et al., 2014; Reid & Brock, 2014; Sakai & Takeuchi, 2000; Watson, Quincey, Carrivick & Smith, 2017; Watson, Quincey, Smith, et al., 2017; Westoby et al., 2020); slope oversteepening, for example from differential melt under the debris (Sakai et al., 1998; Sharp, 1949; Westoby et al., 2020); crevasse opening (Reid & Brock, 2014); undercutting by supraglacial ponds or streams (Moore, 2018; Nicholson et al., 2018); and melt enhancement at pond margins (Miles, Steiner, et al., 2017; Miles, Willis, et al., 2017; Röhl, 2006, 2008; Sakai & Takeuchi, 2000) that may sometimes lead to accelerated steepening from calving (Benn et al., 2012; Immerzeel et al., 2014; Röhl, 2006, 2008).

Contrary to the surrounding debris‐covered ice, ice cliffs are directly exposed to incoming radiation and therefore act as melt “hotspots” (Buri, Miles, et al., 2016; Juen et al., 2014; Sakai et al., 1998). In spite of the small area they occupy, ice cliffs and ponds are responsible for a significant contribution to glacier ablation, and ice cliff melt is estimated to be 3 to 8 times higher than debris‐covered ice melt (Brun et al., 2018; Buri et al., 2021; Immerzeel et al., 2014; King et al., 2020; Mölg et al., 2019; Pellicciotti et al., 2015; Reid & Brock, 2014; Sakai et al., 1998; Thompson et al., 2016). By promoting the backwasting of steep slopes due to enhanced ablation, ice cliffs and ponds influence the morphology of debris‐covered glaciers (Mölg et al., 2020; Watson, Quincey, Carrivick & Smith, 2017) and play a role in their long‐term evolution by increasing their sensitivity to warming (Ferguson & Vieli, 2021). This could, at least partially, explain regional observations of enhanced mass loss of debris‐covered glaciers (Gardelle et al., 2013; Kääb et al., 2012; Ragettli et al., 2016), in spite of the overall melt‐reducing effect of debris cover (Ostrem, 1959). Despite their important role in controlling the long‐term evolution of debris‐covered glaciers, cliffs and ponds are seldom represented in glacier melt models or glacio‐hydrological models. The few models that have tried to account for ice cliffs and ponds use a fixed or linearly derived melt enhancement factor (Hagg et al., 2018; Kraaijenbrink et al., 2017), or arbitrarily reduce the debris thickness (Ferguson & Vieli, 2021; Ragettli et al., 2015). In these models, a fixed cliff area is usually set, which is either the same as the pond area (Kraaijenbrink et al., 2017) or correlated to it (Ragettli et al., 2015). To our knowledge, only two models used cliff outlines manually derived from high‐resolution satellite imagery (Buri et al., 2021; Hagg et al., 2018) and only one study modeled the energy balance of each individual cliff at the glacier scale to quantify their contribution to melt (Buri et al., 2021). This poor representation comes from the difficulty of mapping ice cliffs from remote sensing imagery (Herreid & Pellicciotti, 2018; Kneib et al., 2020), resulting in a very limited knowledge about their distribution, especially over time, as well as their birth and decay mechanisms. While most of the work on ice cliffs has focused on a detailed analysis of a few of these features for an individual site at a single point in time (Anderson et al., 2021; Brun et al., 2016; Buri, Pellicciotti, et al., 2016; Reid & Brock, 2014; Sakai et al., 2002; Steiner et al., 2015; Watson, Quincey, Smith, et al., 2017; Westoby et al., 2020), two studies have looked at ice cliff distribution and evolution over several years for the multiple glaciers of a large catchment (Steiner et al., 2019; Watson, Quincey, Carrivick & Smith, 2017). These two studies offer major advances in understanding cliff dynamics at the glacier scale, showing that there is a high interannual variability in the cliff population. However, they largely ignored the controls or underlying processes of this variability. In both cases, the data used were irregularly spaced in time and still relatively sparse, with at most one image every second year.

As a result, our understanding of the life cycle of ice cliffs, its drivers and its implications for the dynamics of the cliff population of a whole glacier is limited. Ponds and cliffs are often found close to one another, and it has been hypothesized that ponds contribute to the sustainability of cliffs due to their marginal melt effects, although cliffs can also survive for years without being connected to a pond (Brun et al., 2016; Miles et al., 2016; Steiner et al., 2019; Watson, Quincey, Carrivick, & Smith, 2017). Conversely, streams and cliffs may be associated, and it has been suggested that streams meandering across the debris‐covered surface can lead to significant melt (Gulley et al., 2009) and to the formation of supraglacial valleys, or cryo‐valleys, on the sides of which ice cliffs can form (Mölg et al., 2020; Watson et al., 2016). The development of such valleys has only been studied at one site and the length of time over which they evolve is not clear (Mölg et al., 2020). Beyond the supraglacial hydrology, incoming shortwave radiation is the main contributor to the melt of ice cliffs and therefore influences their preferential orientation and survival (Buri & Pellicciotti, 2018). In addition, climatic variability is expected to have an influence on ice cliffs by influencing glacier melt and thus the presence, volume, or discharge of streams and ponds, but also by promoting ice cliff melt and backwasting (Buri, Miles, et al., 2016; Buri, Pellicciotti, et al., 2016; Reid & Brock, 2014; Sakai et al., 1998; Steiner et al., 2015). Surface meltwater and precipitation also determine the debris water content and therefore the debris layers' stability, which has implications for the formation and growth of the cliffs (Moore, 2018). Finally, crevasse opening has been pointed out as one of the possible events triggering the formation of a cliff (Reid & Brock, 2014), which would imply that glacier flow also influences cliff dynamics. However, it is difficult to assess the importance of these individual drivers and their contributions to the cliff dynamics at the glacier scale.

The aim of this study is therefore to understand and model the interannual ice cliff dynamics and how they influence the variability of the cliff population at the glacier scale. Specifically, we aim to (a) quantify the interannual variability of ice cliffs on a feature‐by‐feature and population basis; (b) attribute the observed interannual changes to individual types of cliff change; (c) characterize the stochastic behavior of cliff populations at the glacier scale and develop a model that represents it; and (d) assess the influence of external drivers on the interannual cliff variability.

## 2 Sites and Data

### 2.1 Site Description

To study the interannual variability of ice cliffs and its drivers at the glacier scale, we selected sites in different climatic settings and with different glacier characteristics (velocity, mass balance) but similar size and debris cover stage. Additionally, there needed to be a continuous and relatively long time series of satellite images to map ice cliffs over large portions of the glacier. Upon inspection of the Planet Labs RapidEye, we therefore identified four sites with sufficient suitable data.

**Figure 1:**
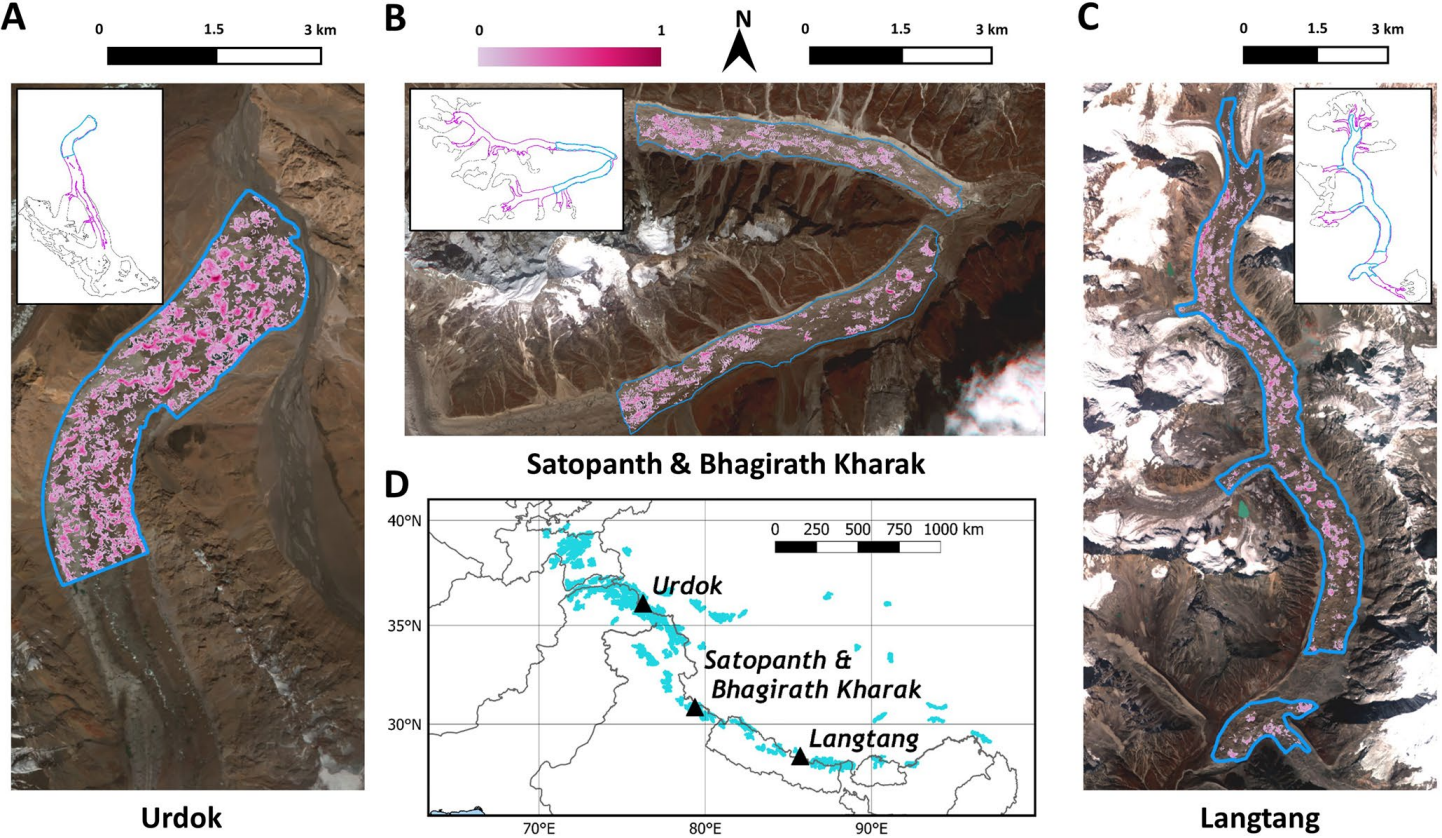
Maps of the four study glaciers and their general location in HMA. Background image is the 2015 RapidEye scene for each site (color composite of bands 4, 2, and 1). The blue outlines correspond to the area of the glacier where the cliffs were mapped (area of interest [AOI]) and the pink colors correspond to the cliff density maps, where transparent color stands for no occurrence of cliffs while 1 corresponds to the presence of cliffs in all the images of the time series (2009–2019 for Urdok and Langtang, 2010–2019 for Satopanth and Bhagirath Kharak). The insets in (a), (b), and (c) show the AOIs (blue), debris‐covered areas from Herreid and Pellicciotti (2020b) (purple), and RGI 6.0 glacier outlines (black) of each site.

We derived the long‐term evolution of ice cliffs for four debris‐covered glaciers located in the Karakoram (Urdok Glacier, Pakistan), Garhwal Himalaya (Satopanth and Bhagirath Kharak Glaciers, India), and Nepal Himalaya (Langtang Glacier) (Figure 1). The glaciers' ablation areas were between 66% and 72% debris‐covered, which corresponds to an advanced stage of debris‐cover (Herreid & Pellicciotti, 2020b; Table 1). For Satopanth the debris is very thick (>1 m) across much of the debris‐covered area (Shah et al., 2019), and this is also thought to be the case on Langtang based on field observations from 2019. To our knowledge, no debris thickness measurements are available for Urdok or Bhagirath Kharak. Previous studies have shown that for Satopanth, Bhagirath Kharak, and Langtang, ponds accounted for 0.6%–2% of the debris‐covered area and cliffs between 3.3% and 9.2% (Kneib et al., 2020; Miles, Willis, et al., 2017; Steiner et al., 2019; Table 1). On Langtang Glacier, it has been observed that cliffs and ponds tend to have a smaller relative area, defined as the ratio between the cliffs or ponds planimetric area and the area of the glacier over which they were mapped, in the dry post‐monsoon than during the wet monsoon season (Miles, Willis, et al., 2017; Steiner et al., 2019). This is consistent with observations made at other HMA glaciers (Watson et al., 2016).

**Table 1:** Glacier Characteristics

Glacier	References	Urdok	Satopanth	Bhagirath Kharak	Langtang
Length (km)	RGI Consortium (2017)	27	15	20	19
Debris cover (% total glacier area)	Herreid and Pellicciotti (2020b)	22	60	41	49
Stage (debris‐covered portion of ablation area)	Herreid and Pellicciotti (2020b)	0.66	0.68	0.72	0.71
Evidence/records of surging (years)	Bhambri et al. (2017)	Yes (1993–1997)	No	No	No
Mean 2009–2018 velocity along centerline in the debris‐covered part (m.yr^−1^)	Gardner et al. (2018)	23.0	14.7	14.7	4.1
Previously reported cliff density (%)	Steiner et al. (2019)	NA	NA	NA	3.4 (±0.9)
	Kneib et al. (2020)	NA	9.2	9.2	3.3
Previously reported pond density (%)	Miles, Willis, et al. (2017)	NA	NA	NA	0.6–2
	Kneib et al. (2020)	NA	0.7	0.7	1.7
Glacier mass balance (m w.e.yr^−1^)	Brun et al. (2017b)	−0.1 (±0.3)	−0.5 (±0.3)	−0.3 (±0.3)	−0.5 (±0.3)

*Note*. Glacier mass balance was obtained by integrating elevation difference data from Brun et al. (2017b) over the entirety of the glaciers and using a value of 850 ± 60 kg.m^−3^ for ice density (Brun et al., 2017b; Huss, 2013).

These four glaciers exhibit long debris‐covered tongues (Table 1) and the terminus positions of Satopanth, Bhagirath Kharak and Langtang have not changed considerably in the past several decades (Nainwal et al., 2016; Wijngaard et al., 2019). These three glaciers show however negative mass balances of −0.5 to −0.3 (±0.4) m w.e.yr^−1^ (Table 1), have relatively low average velocities along the centerline of their debris‐covered area (Table 1), and their mass imbalance therefore translates into stagnating tongues with significant downwasting (Anderson & Anderson, 2016; Ferguson & Vieli, 2021; Rowan et al., 2015). Urdok stands out from these three with its smaller negative mass balance (Table 1), its relatively high velocities (23.0 m.yr^−1^ for 2009–2018 on average) and evidence of a previous surge in the 1990s (Bhambri et al., 2017). Urdok is therefore much more dynamic and in a healthier state than the three other glaciers, despite displaying an extensive debris‐covered area.

### 2.2 Remote Sensing Data

We derived time series of ice cliffs and ponds at our four sites from RapidEye satellite images taken at a yearly interval from 2009 to 2019 (Table S1). The RapidEye images acquired from Planet Labs (Planet Team, 2017) are already atmospherically corrected. They have five spectral bands in the visible and near‐infrared, with a spatial resolution of 5 m. We selected images that were all taken during the post‐monsoon period (end of August to December; Table S1), when stable and dry conditions maximize the chances of acquiring cloud‐free images. No RapidEye images were found in 2009 or 2019 for Satopanth and Bhagirath Kharak or in 2019 for Urdok. For the missing 2019 images, we used PlanetScope images instead (Planet Team, 2017), resampled from 3 to 5 m spatial resolution using a nearest‐neighbor interpolation. Images were selected at a yearly interval in order to ensure similar atmospheric conditions and remove the influence of the seasonal variations of cliffs and ponds (Miles, Willis, et al., 2017; Steiner et al., 2019; Watson et al., 2016). Therefore, we assumed that our observations would be the results of continuous changes from 1 year to the next. Images with snow and shadows from the surrounding topography were avoided as much as possible, but this was not always feasible and we could therefore not delineate ponds and cliffs across the whole debris‐covered area but had to limit the delineation to an area of interest (AOI) for each glacier, defined as the area where the mapping was possible over the full‐time series (Figure 1). Furthermore, we did not apply the mapping over avalanche cones, including, for Langtang Glacier, those originating from the large avalanches triggered after the 2015 Gorkha earthquake in Nepal (Kargel et al., 2016). The final AOIs correspond to 43%, 23%, 40%, and 63% of the total debris‐covered areas (from Herreid & Pellicciotti, 2020b) of Urdok, Satopanth, Bhagirath Kharak, and Langtang, respectively (Figure 1).

### 2.3 Glacier Velocity and Climate Data

To look at the controls of cliff dynamics we accessed the annual velocities for the years 2009–2018 from the NASA MEaSUREs—ITS_LIVE project (Dehecq et al., 2019; Gardner et al., 2018), resampled from 240 to 120 m resolution using the cubic spline interpolation method. For the debris‐covered part of our study glaciers, the reported uncertainty maps from ITS_LIVE displayed very low values (<1 m.yr^−1^) and were thus considered negligible in everything that follows.

We used monthly air temperature and precipitation reanalysis data from the ERA5‐LAND product (Muñoz Sabater, 2019), available in a 0.1° × 0.1° grid. These time series covered our full study period. For each of the study glaciers, we used the ERA5‐Land data from the grid cell covering the center of the debris‐covered area.

## 3 Methods

### 3.1 Image Pre‐Processing

RapidEye images have an expected positional accuracy of less than 10 m according to the product specifications (Planet Team, 2017). Additionally, for each site, the orthoimages were all co‐registered to the initial 2009 image (2010 for Satopanth and Bhagirath Kharak) using the ImGRAFT normalized cross‐correlation algorithm (Messerli & Grinsted, 2015) applied to near‐glacier stable terrain, to ensure the best possible relative positioning.

### 3.2 Mapping

Ice cliffs were mapped manually by one operator in all multi‐spectral images. The mapping was conducted independently for each image. We used shape and color information, as well as local changes in surface motion between two consecutive images as indicators of cliff location (Figure S1). Three automated mapping methods, the Adaptive Binary Threshold, Spectral Curvature, and Linear Spectral Unmixing with scale (Anderson et al., 2021; Kneib et al., 2020), were tested to map the cliffs but the results were not conclusive due to the varying illumination conditions and variable off‐nadir viewing angles resulting in increased shading for some of the scenes compared to the Pléiades and Sentinel‐2 sensors for which the methods above were developed (Kneib et al., 2020; Watson et al., 2018). The ponds were mapped using a fixed Normalized Difference Water Index (NDWI) threshold of 0.1 (Huggel et al., 2002; Kneib et al., 2020; McFeeters, 1996; Watson et al., 2018), and the outlines were improved manually for ice‐covered ponds, which were always a minority.

The data used for the delineation of cliffs are of varying illumination and shading, which can lead to difficulties in identifying features (Kneib et al., 2020; Steiner et al., 2019). We estimated the uncertainty in the cliff and pond relative area obtained from our delineation by comparing our outlines with the consensus outlines produced by three independent operators for six domains of similar sizes ranging between 0.3 and 0.9 km^2^ (two domains on Urdok and Langtang, one on Satopanth and one on Bhagirath Kharak) for the 2011 and 2016 images (Figure S2). For each pixel of the original image, we determined the fraction that was covered by cliff and pond areas as outlined by the three independent operators. We summed the three resulting fraction maps and defined the consensus outlines as the pixels with final values higher than 1.5. The final uncertainties for the cliff mapping were then taken as the mean of the absolute residual values, which were equal to 28% and 41% for the number of cliffs and ponds, respectively, and to 33% and 37% for their respective relative area (Figure S2).

As we are interested in the ice cliff variability, the precision is more important here than the accuracy, meaning that it is more critical to map individual cliff change in time right, rather than counting accurately all the cliffs and ponds in the images. Indeed, we needed to make sure that the observed trends were meaningful. For this, we compared the cliffs and ponds number and relative area obtained by the four independent operators (Operator 4 having mapped the cliffs and ponds in all images) to make sure that there was a good agreement in the resulting changes (Figure S3). The changes derived by Operator 4 from 2011 to 2016 in the validation domains agreed with the ones derived by at least two other operators in 10 cases out of 12 (Figure S3).

### 3.3 Tracking of Cliffs

**Figure 2:**
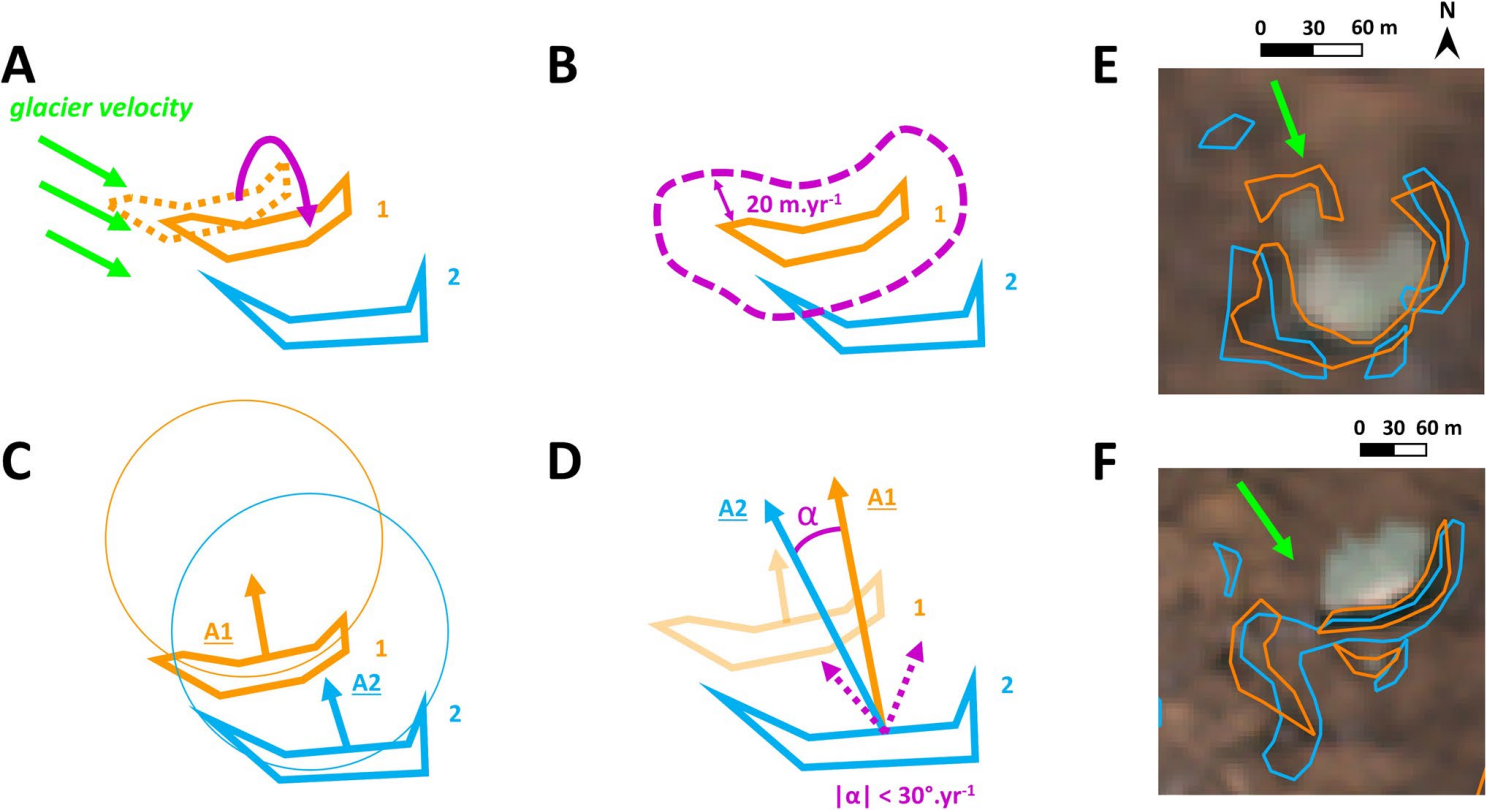
Tracking methodology. In orange are the cliff outlines in year 1 and in blue the cliff outlines in year 2. Panels (a)–(d) represent the different steps of the tracking with idealized outlines. (a) Correction for glacier velocity. (b) Distance check. (c) Aspect retrieval from fitted circles. (d) Mean aspect comparison. Panels (e) and (f) represent real cliff outlines from 2015 (orange) and 2016 (blue) on Langtang. (e) Split and persist events. (f) Merge, birth, and persist events. Background image in panels (e) and (f) is the RapidEye 2015 scene (color composite of bands 4, 2, and 1). The green arrows represent the glacier flow direction.

Once all the cliffs had been outlined in the images, we automatically tracked the evolution of each individual cliff from one image to the next, using a new algorithm developed ad hoc to directly compare the characteristics of the cliffs in consecutive images (Figure 2). For two consecutive images, image 1 and image 2, we accounted for glacier surface velocity using the annual ITS_LIVE data (Dehecq et al., 2019; Gardner et al., 2018) to shift the position of the cliffs in image 1 to compensate for glacier motion (Figure 2a). We then compared the cliff outlines of image 2 with the shifted cliff outlines of image 1. If the distance between the cliffs was less than 20 m in a year (Figure 2b) and the aspect difference less than 30° in a year, modulus(180°) (Figure 2d), we considered the cliffs to be the same, with 1‐year difference. The 20 m.yr^−1^ threshold is conservative relative to observed cliff backwasting rates that are usually less than 15 m.yr^−1^ (Brun et al., 2016, 2018; Buri, Miles, et al., 2016; Han et al., 2010; Mölg et al., 2019; Reid & Brock, 2014; Steiner et al., 2015; Watson, Quincey, Carrivick & Smith, 2017) and therefore also accounts for co‐registration and surface velocity uncertainties. We did not have any Digital Elevation Model (DEM) for the RapidEye images, so the cliff aspect was estimated by fitting a circle to the cliffs' vertices using a quasi‐Newton optimization method (Umbach & Jones, 2000; Figure 2c). We approximated the aspect of each pixel as the direction of the vector starting at the center of the pixel and finishing at the center of the circle. We calculated the aspect of the cliff as the circular mean aspect of its pixels (Figure 2c). Therefore, for the aspect difference, it was necessary to take modulus(180°) to account for straight cliffs that can see their mean aspect change by ±180° from 1 year to the next. For cliffs with a standard deviation of the aspect greater than 45°, we only compared the aspect of the pixels less than 20 m away to identify nearby circular cliffs as independent from one another. These tracking parameters (Table S2) were calibrated against manual tracking of cliffs between the 2009 and 2010 Langtang scenes and validated for a randomly selected year at each of the three other sites. With these parameters, the automated and manual tracking agreed for 96.4% of the cliffs in the initial image for Langtang, and 90.2%, 91.7%, and 90.0% for Urdok, Satopanth, and Bhagirath Kharak, respectively.

Based on the tracking results, we could determine the evolution of each cliff in time, but also the number of cliffs that appeared (birth events) or disappeared (death events) every year. We also quantified the split events, when one cliff splits into two cliffs or more (Figure 2e), and merge events, when two or more cliffs merge into one (Figure 2f). Cliffs were also observed to split and merge at the same time, resulting in a mix event. The most common event on the other hand was a cliff remaining a single cliff but changing shape and/or size, which we describe here as a persist event (Figures 2e and 2f).

## 4 Observational Evidence of Cliff Dynamics

### 4.1 General Dynamics of Cliffs and Ponds

**Figure 3:**
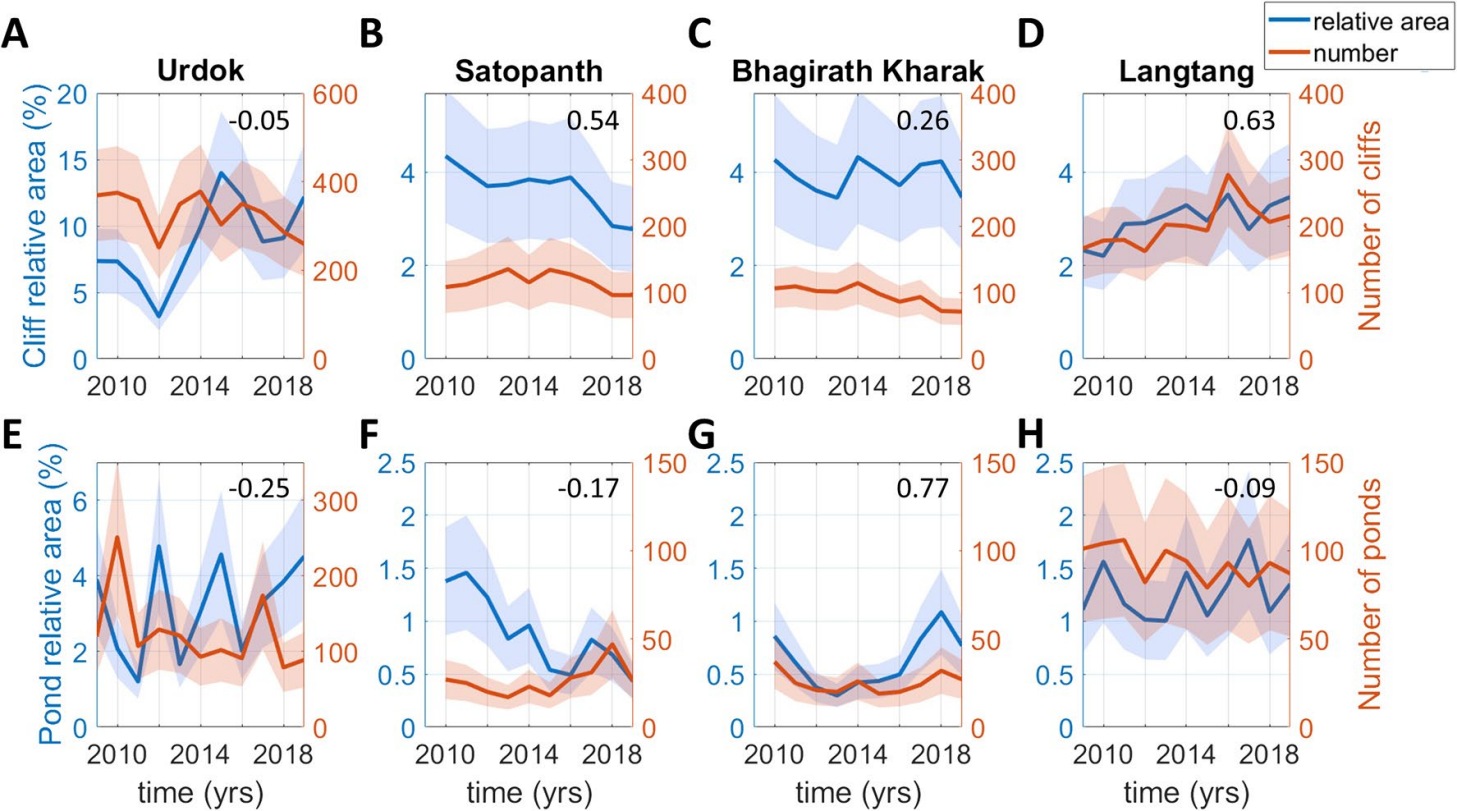
Time series of cliffs and ponds number and relative area. The *y*‐axis scales are different for Urdok and the three other glaciers. The shaded areas correspond to the uncertainty range calculated in the methods. The numbers in the upper right‐hand corner of the plots are the Pearson's correlation coefficients between the relative area and number time series.

The cliff relative area and the cliff number change considerably from one year to the next (Figure 3). These two variables are mostly uncorrelated, except for Langtang and to some extent for Satopanth. The number of ice cliffs remains centered around 328 for Urdok (Figure 3a) and 116 for Satopanth (Figure 3b), while it shows a small decrease for Bhagirath Kharak from an average of 106 to 84 cliffs between the first and second half of the study period (Figure 3c) and a small increase for Langtang, from 177 to 221 (Figure 3d). Although the number of cliffs remains relatively constant, Urdok experiences a large increase in cliff relative area between the years 2012 and 2015 from 3.2% to 14.0%. The cliff relative area on Urdok is also generally higher than the cliff relative area of the other three glaciers. The cliff relative area changes every year on Satopanth, Bhagirath Kharak, and Langtang centering around 3.6%, 3.9%, and 3.0%, respectively, with a slight decrease for Satopanth from 3.9% to 3.3% and a slight increase for Langtang from 2.7% to 3.2% on average between the first and second half of the study period.

For all four glaciers, the cliff relative area is higher than the pond area by a factor ranging between 2.4 for Langtang and 7.3 for Bhagirath Kharak over the whole time series. For each glacier, the pond relative area and number appear to be uncorrelated with the cliff area and number, except for Satopanth where both pond and cliff relative area exhibit a decreasing trend. The pond relative area is much higher for Urdok (average value of 3.2%) than for the other glaciers, for which the average pond relative area is between 0.6% and 1.3%. The pond relative area on Urdok also has very strong variations and can more than double in a single year (Figure 3e). The number of ponds is not correlated with the pond relative area except for Bhagirath Kharak. The number of ponds also remains relatively constant over time, except for Urdok, where there are also strong variations at the beginning of the study period.

### 4.2 Contributions to the Cliff Dynamics

**Figure 4:**
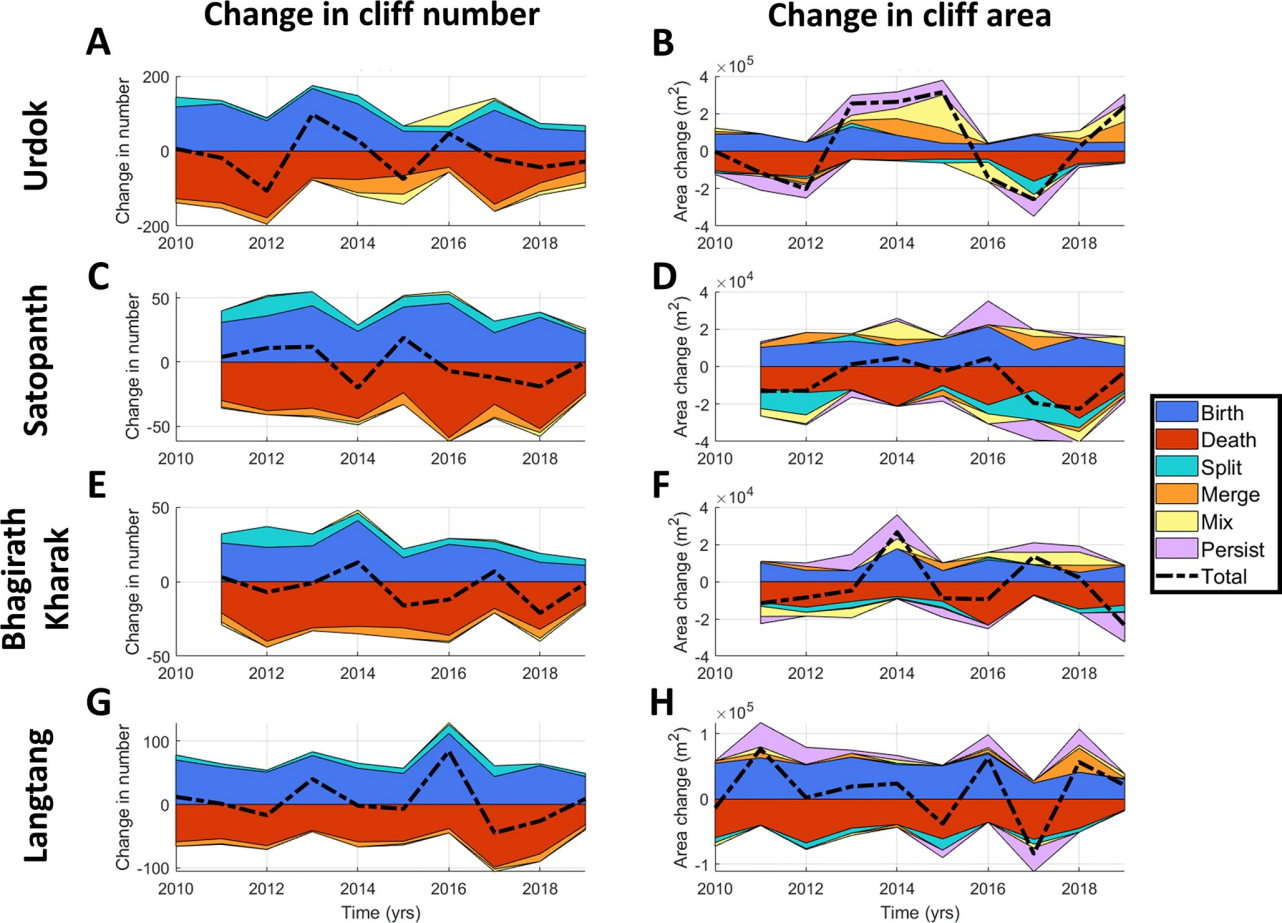
Net contribution of different events to the general evolution of ice cliffs. The left panels show the change in cliff number and the right panels show the change in cliff area. For comparison, the mean cliff number (area) is 328 (6.8 × 10^5^ m^2^), 116 (1.5 × 10^5^ m^2^), 95 (1.2 × 10^5^ m^2^) and 201 (3.3 × 10^5^ m^2^) for Urdok, Satopanth, Bhagirath Kharak, and Langtang, respectively. Note that the color envelopes are not overlapping but stacked on top of one another.

The tracking of the individual cliffs allowed us to count the birth, death, split, merge, and persist events over time (Figures 4 and S4). By taking into account the cliff area before and after each event, we also derived the net and total area change related to each event for the whole cliff population (Figures 4 and S4). This enabled us to directly derive the contributions of different events to the changes in cliff number and area.

For all four glaciers, the change in cliff number is centered around zero and the standard deviation varies between 10% and 20% of the total cliff number. For the four glaciers, the change in number is driven by the birth and death events. Split and merge events contribute respectively positively and negatively to the change in number, mix events can cause changes in both directions (Figure 4a), and persist events do not contribute to the change in number but can contribute to the change in area. Split, merge, and mix events only contribute to less than 10% of the change in number, except from 2014 to 2016 for Urdok where they contribute up to 40% of the change. In most cases, except for some years in Urdok and Bhagirath Kharak, an increase in cliff number is followed by an increase in death events. This is consistent with what we observe when comparing the number of death events and the total cliff number one year before (Figure S5), which shows that death events tend to compensate for a higher‐than‐average number of cliffs.

Changes in cliff area are mostly positive for Urdok, and negative for Satopanth, thus highlighting an increase and a decrease in cliff area, respectively. These changes are centered around zero for Bhagirath Karak and Langtang (Figure 4). Their standard deviation varies from 7% of the cliff relative area for Satopanth to 45% for Urdok. For all glaciers, there is a significant contribution of birth and death events to changes in cliff relative area, and peaks in cliff relative area change usually correspond to peaks in birth events and/or lower death rates (Figure 4). These peaks in the contribution of birth events to cliff area are amplified by the contribution of split, merge, mix, and persist events, which usually contribute in the same direction, either positively or negatively (Figure 4). Of all these events, the persist events are usually predominant, even in terms of net contribution, but the mix or split events can also contribute considerably in some years, especially at Urdok and Satopanth.

### 4.3 Cliff Size and Pond Influence

For the four study sites, we find that the cliff size follows a lognormal distribution (Figure S6). This lognormal distribution spans 2 orders of magnitude, from 100 m^2^, which corresponds to four RapidEye pixels and can therefore be considered as the cliff detectability threshold, to ∼10,000 m^2^ (400 pixels), and even to more than 20,000 m^2^ (800 pixels) for some cliffs in Urdok (Figure S6). The size of new cliffs or dying cliffs also follows a lognormal distribution with a similar standard deviation to the persisting cliffs, but they are generally smaller, with the mean of the lognormal distribution around 400–500 m^2^ for Urdok and Langtang, 200–300 m^2^ for Satopanth and Bhagirath Kharak, while it is between 500 and 800 m^2^ for the persisting cliffs (Figure S6). As a result, the cliffs that are about to die, or that are less than 1‐year old, tend to be smaller than other cliffs.

The size of splitting, merging, and mixing cliffs also follows a lognormal distribution (Figure S7). The size distribution of merging cliffs is very similar to that of the persisting cliffs, which means that any cliff can be involved in a merge event, while the cliffs resulting from a merge event are larger than the norm. This is the contrary for split events, in which the cliffs that split are generally larger than normal but the cliffs resulting from split events follow the same size distribution as the total population (Figure S7). For the mix events, the size distribution of cliffs before and after is more or less the same and similar to that of the total cliff population with some variations due to the small number of events relative to the persist events.

For split, merge, mix and persist events, the relationship between the sum of cliff sizes before (*S*_*i*_) and after the event (*S*_*f*_), is given by the area ratio α:

(1) $$\alpha \;=\;\frac{S_{f}}{S_{i}}.$$

Our results show a large spread of *α*, which usually follows a lognormal distribution centered around 1, and range from 0.1 to 10 for the most extreme area changes (Figure S8). For persist events, this area ratio is dependent on the cliff initial size, with a tendency for smaller cliffs to increase in size while the larger cliffs tend to decrease in size (Figure S8). Such a trend is difficult to observe for other events, which occur less often than persist events.

Ultimately, the presence of ponds also has an influence on the birth and death events. For Urdok, Satopanth, and Bhagirath Kharak, 10%–35% of all the cliffs have an attached pond, and for Langtang, this value varies between ∼30% and ∼50% (Figure S9). At all sites, the proportion of new or dying cliffs with an attached pond (less than 10 m away) is consistently lower than for the whole population, and can be as low as 10%–12% for Langtang and Urdok, 0%–2% for Satopanth and Bhagirath Kharak (Figure S9). Urdok is the site where the difference is less visible.

## 5 Stochastic Modeling of Cliff Dynamics

Based on our observations from the tracking of ice cliffs we implemented a stochastic birth‐death model to represent the cliff population dynamics and the interannual changes in cliff number and area for each glacier. We implemented two models, one that is purely stochastic and all events occur at random, for which we considered the cliff populations to be closed systems with no influence from external drivers, and the second where we included the influence of air temperature, precipitation, pond area, and surface velocity on the birth and death rates and area ratios of persist events. In this second model, we kept the mix, split and merge events as stochastic events since they represent a minority of events and the influence of the external drivers was unclear.

### 5.1 Stochastic Birth‐Death Model

The purpose of a purely stochastic model is to describe the natural internal variability of a system given by randomly occurring events in time. A birth‐death model is a basic type of model commonly used in ecology or epidemiology to study the demography of a population and to provide information on the probability distributions of the number and characteristics of individuals (Bailey, 1968; Kendall, 1948). Such models have also been used in the simulation of the birth and death of rainfall cells (e.g., Paschalis et al., 2013). Birth‐death models characterize the evolution of a population following the underlying equation:

(2) $$\frac{dP}{dt}=B\left(t\right)-D\left(t\right),$$

where *P*(*t*) is the number of individuals (cliffs) in a given year, *B*(*t*) and *D*(*t*) are the birth and death rate in cliffs/year, respectively. In the case of ice cliffs, we include merge, split, and mix events, and the governing equation for the stochastic cliff population dynamics becomes:

(3) $$\frac{dP}{dt}=\;B\left(t\right)-D\left(t\right)+S\left(t\right)-M\left(t\right)+Mi\left(t\right),$$

where *S*(*t*), *M*(*t*), and *Mi*(*t*) are the split, merge, and mix rates in cliffs/year, respectively. *Mi*(*t*) can be positive or negative while the other terms are all positive. All the terms are described by their probability density functions (mean and variance) estimated from data. There is a linear dependency between the death rate and the population size (Figure S5):

(4) D(t+dt)=aP(t)+b+ξ,

where *a* and *b* are estimated from the death rates related to the total population and *ξ* is a stochastic noise term (Supporting Information S1).

Equation 3 is solved in a time‐stepping manner with *dt = 1 year*, by drawing the terms *B*, *S*, *M*, *Mi*, and *ξ* from their respective distributions, starting at time zero with a given initial cliff population. Each individual cliff in the population is assigned to undergo a death, mix, merge, split or persist event based on the probability of individual events in every year, its size and the size distribution of dying, mixing, merging, splitting, and persisting cliffs (Supporting Information S1, Model Description: 3. Cliff selection). The cliffs are tracked individually in the model and their size is updated at each time step based on the area ratio distribution of the merge, split, mix, or persist event that they undergo. New cliffs are attributed an initial size which is drawn randomly from the size distribution of new cliffs, and dying cliffs are removed from the cliff population. The implementation of the model is described in detail in the Supporting Information S1 (Model Description; Figure S10 and Tables S3 and S4).

Since the processes controlling the size changes of the cliffs are also stochastic for each event, the cliff area *A*(*t*) can be written following a similar equation as Equation 3:

(5) $$\frac{dA}{dt}=\;B_{A}\left(t\right)-D_{A}\left(t\right)+S_{A}\left(t\right)+M_{A}\left(t\right)+Mi_{A}\left(t\right)+Pe_{A}\left(t\right),$$

where *B*_*A*_ *, D*_*A*_ *, S*_*A*_ *, M*_*A*_ *, Mi*_*A*_ *,* and *Pe*_*A*_ are the changes in size from the birth, death, split, merge, mix, and persist events which can be positive or negative, except for *B*_*A*_ and *D*_*A*_ which are always positive.

Note that if we combine Equations 3 and 4 in a steady‐state case ((dP/dt)=0and(dA/dt)=0), the mean changes from the different events compensate each other and force the mean population $\bar{P}$ to a given value that is independent of the initial conditions:

(6) $$\bar{B}-\left(a\;\bar{P}+b\right)+\bar{S}-\bar{M}+\bar{Mi}=0.$$

Similarly for the cliff relative area (Equation 5).

In this first version of the model, the probability distributions of the different terms of Equations 3 and 5 are fixed in time and in this case *P*(*t*) will converge in time to its steady‐state value $\bar{P}$. However, these probability distributions can also be time‐dependent with individual annual rates as a function of external driving forces.

### 5.2 Influence of External Drivers

In the second version of the model, we take into account external drivers and their influence on the cliff population dynamics. These external drivers are the monthly air temperature and precipitation from ERA5‐Land (Muñoz Sabater, 2019) averaged over the summer months (June–September), the average annual glacier velocity and change in velocity over the centerline of the debris‐covered part and the AOI from the ITS_LIVE velocity data (Dehecq et al., 2019), and the total pond area and change in pond area (Tables S5–S8). We relate cliff population dynamics and external drivers using a stepwise multivariate regression for the birth rate, relative death rate (defined as the ratio of death events and the total number of cliffs at the previous time step), and the parameters of the size‐dependent mean area ratio of persist events (Supporting Information S1, Stochastic model description, Tables S5–S8). For each of these regressions, we use a linear model with an intercept and a linear term for each predictor. The predictors are added using stepwise regression if adding them increases the adjusted correlation coefficient adj‐*R* ^2^ value by more than 0.1, which guarantees that the new terms improve the model more than they would be expected by chance (Miles, 2014). This multivariate regression is applied to the whole time series from 2009 (2010 for Satopanth and Bhagirath Kharak) to 2018, since we only had velocity data until that year. We then account for the external drivers in the model by rewriting the birth rate, relative death, and area ratio parameters at each time step Param_*j*_(*t*) as a function of the eight external drivers *D*_*i*_ using the coefficients *a*_*i,j*_ and RMSE from the multivariate regression:

(7) $${\text{Param}}_{j}\left(t\right)\;=a_{0,j}+\sum_{i=1}^{8}\delta_{i,j}a_{i,j}D_{i}\;+\lambda_{j}\left(t\right)\;,$$

where $\lambda_{j}\left(t\right)$ is a stochastic term drawn at each time step from a discrete normal distribution of mean zero and standard deviation equal to the RMSE of the multivariate regression. $\delta_{i,j}$ is equal to zero or one depending on the inclusion or exclusion of the external driver in the multivariate regression (Tables S5–S8).

### 5.3 Modeling Results

**Figure 5:**
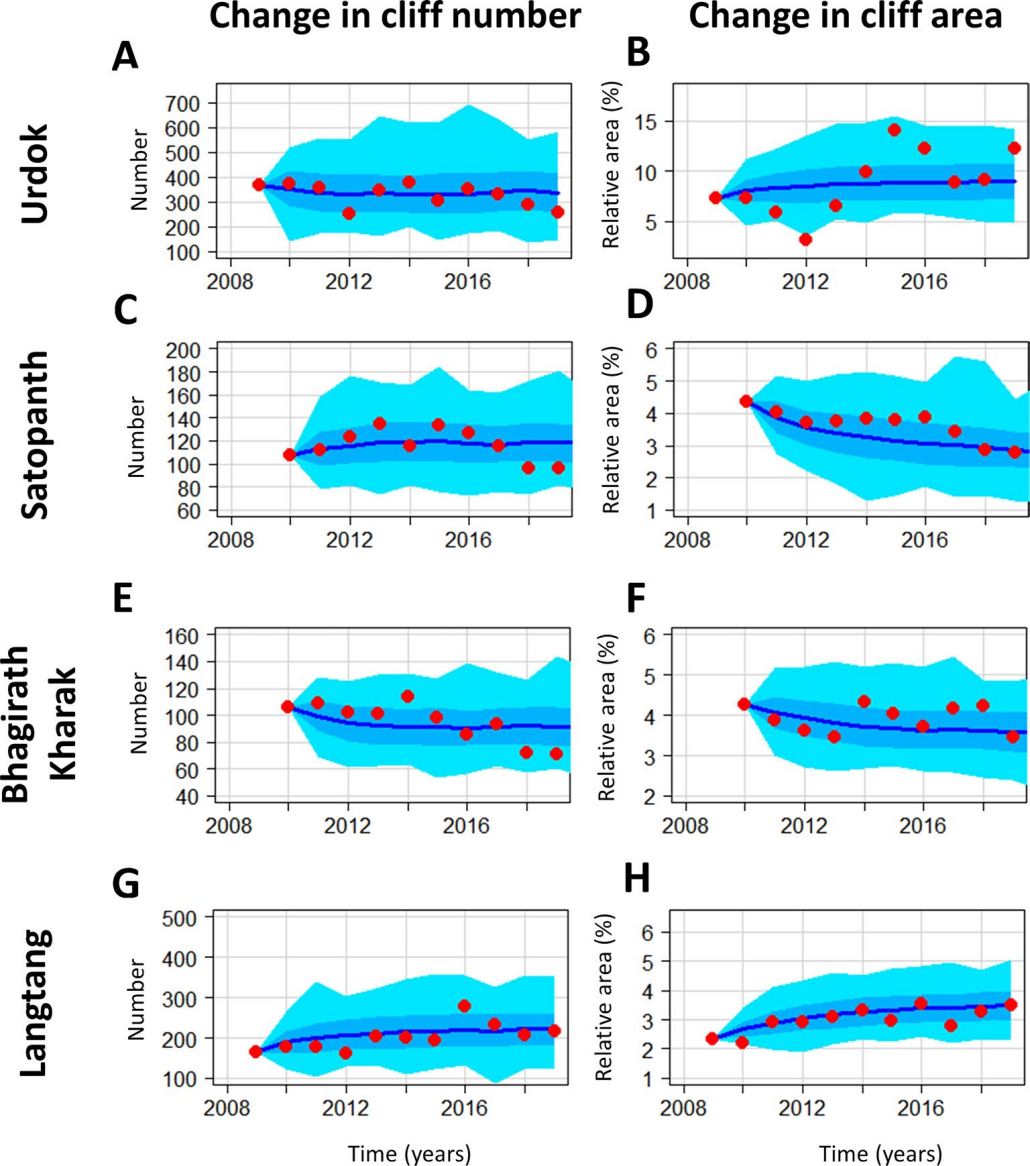
Outputs from the stochastic model for the number of cliffs and their relative area. The model was run 200 times from 2009 (2010 for Satopanth and Bhagirath Kharak) to 2019. Parameters were estimated over the full‐time series. The red dots correspond to the observations from the mapping. The dark blue line corresponds to the average values of the 200 simulation runs. The mid‐blue area represents the standard deviation of the runs and the light blue area the maximum and minimum values.

We apply the stochastic birth‐death model (without external drivers, Section 5.1) to the cliff population of the four sites for the duration of the observation time series (Figure 5). The model parameters are estimated from the entire study period, and we run the model 200 times for the period 2009–2019 starting from observed initial conditions in 2009 to quantify the probability distribution of cliff number and area over time. The model converges rapidly and there are no significant changes in mean or standard deviation after more than 200 simulations (Figure S11). The mean cliff number and area both converge to steady states within the first 10 years of the simulations (Figure 5), and the standard deviation range and maximum spread are also stable in time after this point (Figure S12). The results obtained by calculating the parameters over the full‐time series are similar to those obtained when calculating the parameters over the first 5 years, even though the fit is less good, especially for the last years at Satopanth and Bhagirath Kharak (Figure S13), and are independent of the initial conditions (Figure S14). All the observations are within the modeled range of values except for year 2012 for the cliff relative area in Urdok (Figure 5). This variability range is characterized by a standard deviation that is between 12% and 20% of the mean value after 10 years for both cliff relative area and number. This means that the observed variability in cliff properties (number and area) in time can be explained by a model in which cliff‐forming and destruction processes are completely random.

The stepwise multivariate regression applied to the four glaciers shows that the most significant drivers for the birth rates are the total pond area and the change in pond area (Table S5). They are the first predictors selected in the stepwise multivariate regression for Urdok, Satopanth, and Bhagirath Kharak. For Langtang, the air temperature is more significant but the multivariate regression at this site is not statistically significant according to its high *P*‐value (Table S5). Similarly, the glacier velocity and velocity change in the AOI or across the whole debris‐covered area are the most significant drivers for the relative death rate and area ratio for the sites where the multivariate regression is statistically significant (Tables S6–S8). All the tested drivers contribute significantly to a few model parameters at different sites except for precipitation that has no significant contribution (Tables S5–S8).

**Figure 6:**
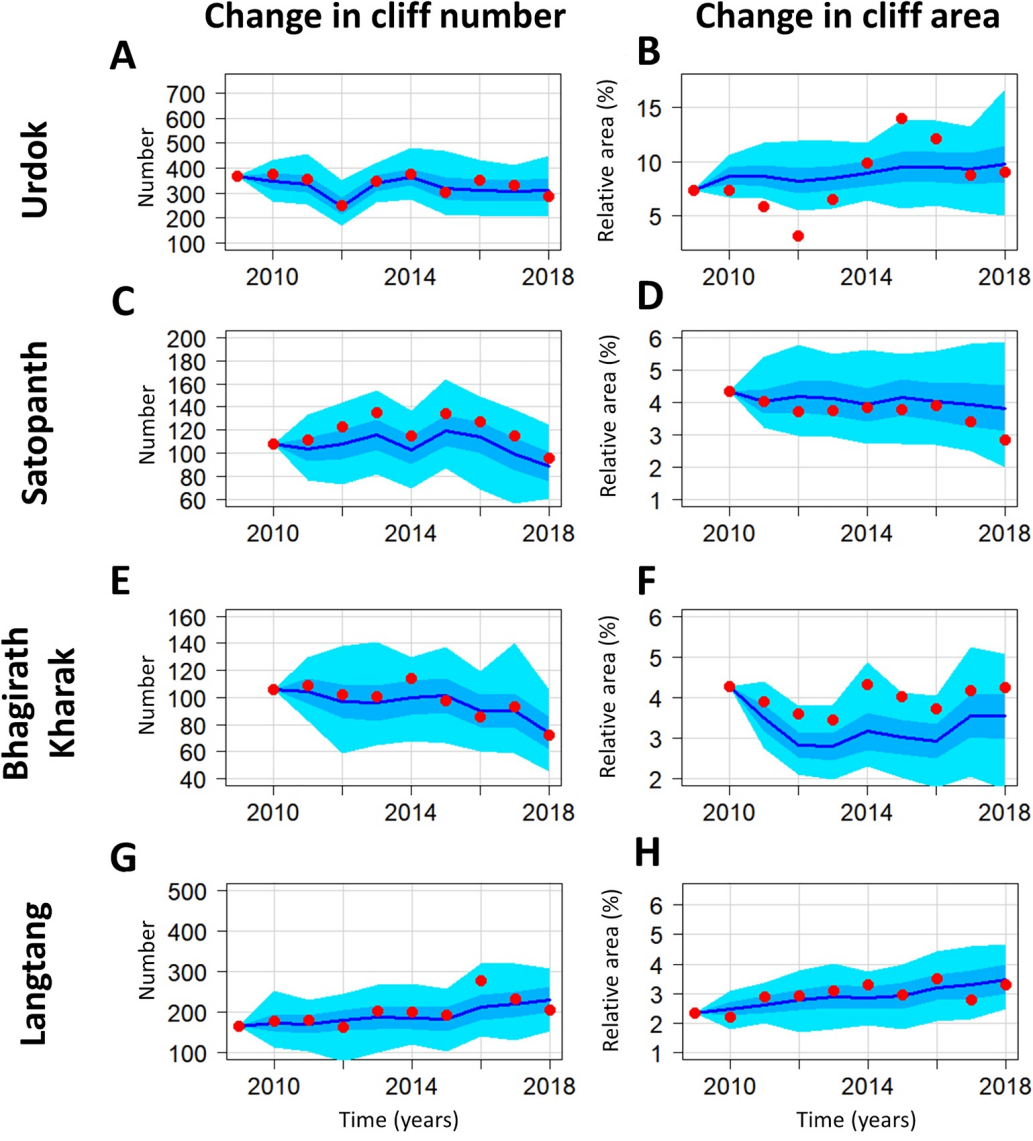
Outputs from the stochastic model with external drivers for the number of cliffs and their relative area. The model was run 200 times from 2009 (2010 for Satopanth and Bhagirath Kharak) to 2019. Parameters were estimated over the full‐time series. The red dots correspond to the observations from the mapping. The dark blue line corresponds to the average values of the 200 simulation runs. The mid‐blue area represents the standard deviation of the runs and the light blue area the maximum and minimum values. The *y*‐axis scales are the same as in Figure 5.

When applying the model accounting for external drivers, we find that the mean cliff number and relative area do not converge but rather follow similar annual variations to the observations (Figure 6). The variability range is smaller than for the purely stochastic model but all the observations fall within the model minimum and maximum values and most of them are within the standard deviation bounds. For Urdok, however, the abrupt increase in cliff relative area between 2012 and 2015 is still not captured well while the cliff relative area is underestimated on average for Bhagirath Kharak (Figures 6b and 6f). This means that external driving variables in the stochastic model are able to reduce uncertainties, better match interannual variability in observations, and in some cases identify situations where the random model fails.

## 6 Discussion

### 6.1 Mapping and Tracking Limitations

For the cliff mapping, we used RapidEye data from Planet Labs (Planet Team, 2017) because of their high spatial resolution (5 m) and relatively long time series (more than a decade of images freely available with an academic license). These are however georectified images without a contemporaneous DEM, so no mapping approach based on slope (Herreid & Pellicciotti, 2018; Reid & Brock, 2014) could be used in a multitemporal sense. We tested different multispectral approaches, namely the adaptive brightness threshold, the spectral curvature, and linear spectral unmixing with scale (Anderson et al., 2021; Kneib et al., 2020), but the results were not satisfying and consistent enough for this sensor, due to the varying illumination conditions and increased shading for some of the scenes compared to the Pléiades and Sentinel‐2 sensors for which the methods above were developed (Kneib et al., 2020; Watson et al., 2018). Despite the important effort required and the possible operator bias (Herreid & Pellicciotti, 2018), we thus favored the use of manual delineation of cliffs for this study. For ponds, we used an NDWI threshold since this approach had already been validated for pond mapping with RapidEye data (Watson et al., 2018). Some manual improvement was still necessary for ice‐covered ponds but the time investment was minimal compared to the manual delineation of all the cliffs. In the future, the use of an automated approach for cliffs using high‐resolution sensors with a high overpass rate to minimize shading such as PlanetScope will likely enable the study of longer time series and on a seasonal time scale.

The main uncertainties from the cliff mapping come from the operator bias (Figure S2) and the change in illumination and shadowing from image to image, especially because we did not have access to any high‐resolution slope data, which can help discriminate some cliffs (Herreid & Pellicciotti, 2018; Steiner et al., 2019). For the same reason, we could not calculate the cliff slope and aspect and only used ice cliff planimetric area to calculate the cliff relative area. The manual mapping was greatly improved by comparing sequential images. Disruptions in glacier surface motion were thus anticipated to be caused by backwasting cliffs or draining or filling ponds, which helped to constrain the mapping. Accounting for these disruptions in glacier surface motion was performed manually in this study but is a promising method to map cliffs and derive their backwasting rates in an automated way (Altena & Kääb, 2020). Furthermore, comparing the outlines with those derived by independent operators for six different domains across six different images enabled us to constrain the uncertainties from the mapping (Figure S2). Ultimately, reducing the uncertainties from the mapping requires automated approaches and higher resolution images (Kneib et al., 2020; Salerno et al., 2012). Cliffs and ponds can be mapped at resolutions coarser than 5 m but this results in higher uncertainties and increases the detectability threshold to only map the larger features (Herreid & Pellicciotti, 2018; Kneib et al., 2020; Miles, Steiner, et al., 2017; Watson et al., 2018). This is especially true for ice cliffs, which have a more elongated shape and can be very steep, thus in general, the finer the spatial resolution, the better (Brun et al., 2018; Immerzeel et al., 2014; Kraaijenbrink, Shea, et al., 2016).

The tracking disagreements between the manually and automatically tracked cliffs were 10% at most and occurred mostly for cases of small cliffs, for which the aspect was difficult to determine, or cliffs with a sharp aspect discontinuity (e.g., Figure 2f). In such cases, it was difficult to tell which of the manual or automated tracking was correct, and since there were less than 10% of disagreements, we considered the uncertainties from the tracking to be negligible compared to the mapping uncertainties. Having an accurate DEM for each image would have reduced the tracking error considerably by eliminating the cases described above (Steiner et al., 2019). Furthermore, the mean aspect of cliffs can change considerably from one year to the next, so we do not recommend the application of this tracking algorithm for intervals longer than a year.

For this study, which focused on the cliff number and relative area variability, the precision and consistency of the mapping were actually more important than the accuracy of the outlines. The agreement between the four independent operators in the trends of cliff area and the number that they derived independently between 2011 and 2016 for six validation domains (Figure S3) shows that while there were disagreements in the actual values, the trends were mostly similar. Therefore, we would expect that if another operator were trying to reproduce the same experiment, the absolute numbers may disagree with the ones from this study, but the variability and general patterns and therefore the results would be the same.

We also note that the AOIs of the four studied glaciers did not cover the full extent of the debris‐covered areas due to snow, shadows, and avalanching (Figure 1), and were mostly limited to the lower portions of the glaciers, where we expect thicker debris and more stagnant ice (Anderson & Anderson, 2018). This could potentially bias these observations toward particular cliff‐types influenced more by ponds than supraglacial streams, and less by ice dynamics. Similarly, the differences in relative coverage of the AOIs on the different glaciers could influence some of the relative changes observed, which needs to be taken into account in the interpretation of the results.

### 6.2 Life Cycle of Ice Cliffs

The systematic mapping of ice cliffs and ponds at annual intervals in the post‐monsoon season and for the same AOI highlights the high variability of these features from one year to the next (Figure 3). This has implications for the melt of debris‐covered glaciers, while from a process understanding standpoint it informs about the rates of changes of the glacier surface. This interannual variability had been investigated before (Steiner et al., 2019; Watson et al., 2016; Watson, Quincey, Smith, et al., 2017; Miles, Willis, et al., 2017) but never in such a consistent way. Our results show that the cliff relative area can change regularly by typically 20% from one year to the next, and the pond relative area by more than 40%. In this regard, Urdok is a special site that exhibits even larger variations (up to 100% changes in cliff relative area and 300% changes in pond relative area). As cliffs and ponds are major contributors to the mass balance of debris‐covered glaciers and enhance melt by a factor of 3–8 relative to the surrounding debris‐covered ice (Brun et al., 2018; Buri et al., 2021; Immerzeel et al., 2014; Juen et al., 2014; King et al., 2020; Miles et al., 2018; Mölg et al., 2019; Reid & Brock, 2014; Thompson et al., 2016), these results highlight the need to take into account the variability of these features in glacier melt models.

Interannual ice cliff variability is extreme at the feature scale, with typically 15%–30%, and in some years up to 50%, of new cliffs forming every year (Figure 4). The dynamics vary from cliff to cliff, with some cliffs observed in only a single year (usually the smallest ones), and others persisting for the whole study period. The evolution of a cliff is partly constrained by the presence or absence of a pond (Figure S8). The fact that at all sites the proportion of pond‐associated cliffs is greater for persisting cliffs than for dying or new cliffs implies that association with a pond is indeed a key factor promoting cliff longevity (Brun et al., 2016; Buri, Miles, et al., 2016; Miles et al., 2016; Watson, Quincey, Carrivick & Smith, 2017). Ponds encourage cliff persistence, and pond drainage can be a precursor to cliff death. Fluctuations in the ponded area have a significant influence on the birth rate of the Urdok and Satopanth cliffs and the relative death rates of the Urdok cliffs (Tables S5–S6). The negative relationship between the cliff birth rate and pond area for Urdok and Bhagirath Kharak (Table S5) could indicate that draining ponds wash away the debris, leading to cliff birth. However, despite this control for individual cliffs, there is no clear relationship between cliff and pond relative area at the scale of an individual glacier (Figure 3) because the ponds only affect less than 50% of the cliff population (Figure S9). Therefore, at the glacier scale, the pond influence is muted by other factors and their contribution to cliff persistence is not always significant. Cliffs and ponds also evolve on different time scales, with ponds having much stronger seasonal variations than cliffs (Miles, Willis, et al., 2017).

The size of the cliff also plays a role in its evolution. We found that cliffs tend to follow a lognormal distribution with regards to their size, which is consistent with what has been found on Khumbu Glacier (Watson, Quincey, Carrivick & Smith, 2017). Some studies using semi‐automated mapping found that the number of cliffs or ponds on a glacier increases exponentially as size decreases (Kneib et al., 2020; Miles, Willis, et al., 2017), which could mean that there is an observational bias for mapping only the large features relative to the sensor resolution (Salerno et al., 2012), but this would need to be confirmed by very high‐resolution mapping from field observations and UAV flights. New cliffs and dying cliffs are in general smaller than the other cliffs, even if there are exceptions. Interestingly, we also found that the area ratio of persist events decreases with the initial cliff size (Figure S7). However, these observations are likely biased by the fact that we cannot detect ice cliffs smaller than a few pixels (<100–200 m^2^), which means that what we count here as birth or death events may just be persist events, but involving features that have passed beyond the satellite images' limit of detectability. The area ratio of persist events is centered around 1 but there are cases where the cliff size is multiplied or reduced by a factor up to 10, which can happen when the cliff expands laterally on the nearby slopes. This expansion or reduction probably depends on the stability of the debris on an ice slope, which is linked to the slope angle but also to the debris water content and the presence of a pond or stream at the base of the slope (Moore, 2018).

Merge, split or mix events also contribute to the interannual variability of the cliff population, especially for the change in cliff relative area. Their contribution to cliff area change follows the same general pattern as the contribution of persist events (Figure 4), which means that while these events are mostly stochastic and, for merge and mix events, dependent on the local cliff concentration, at the glacier scale their contribution to the cliff area change has the same drivers as the persist events.

All these events contribute to increasing the interannual variability of the cliff population, except for death events, which to some extent compensate for strong variations in cliff number and as a result, relative area. Indeed, at all sites, an increase in the number of cliffs usually results in an increase in the number of death events the year after. Such feedback is the basis of most birth‐death models in a closed system with limited resources (Bailey, 1968; Kendall, 1948).

### 6.3 Controls on Ice Cliff Variability

Determining the controls of the variability of the cliff population is important to understand the observed patterns and relate this to processes happening at the glacier surface, but also in a broader sense to understand whether these variations are purely stochastic or on the contrary are representative of a particular glacier state or evolution. The stochastic model runs provide new insights into the natural internal variability of the cliff population at the glacier scale based on the observed variability of the system. This gives a first approximation of the system bounds in the long term, assuming that the glacier surface does not undergo major changes. Our stochastic model outputs a distribution of cliffs that depends on the initial conditions for the first few years but rapidly converges to a steady state that depends on the parameters of the different events and that has an internal variability that is proportional to its mean value. We estimated the parameters over a period of 10 (Figure 5) and 5 years (Figure S13) and obtained similar results despite a less good fit in the second half of the time series when the parameters were estimated over the first 5 years, which shows that the variability of the cliff populations only changed marginally over our observation period. Assuming that the general climatic and glaciological conditions encountered persist, the results of the model inform us on the long‐term variability of the cliff populations of these four glaciers (Figure S12).

The stochastic assumption enables us to calculate the internal variability of the system but does not tell us if this variability can be influenced by external drivers. Adding the influence of external drivers in the model reduces the variability and improves the fit with the observations, which shows that these external drivers do have an influence on the variability of the system. The results from the multivariate regression help explain some of the observed variability and link it with climate, glacier velocity, or pond evolution. None of the tested variables stands out as a principal driver and all contribute to some extent to the observed changes. They are, however, all related to the melt at the surface of the glacier, since climate variables influence melt and this melt will increase the amount of water circulating at the surface of the glacier via ponds and streams, while enhancing basal sliding and therefore glacier velocity (Kraaijenbrink, Meijer, et al., 2016; Yang et al., 2020).

**Figure 7:**
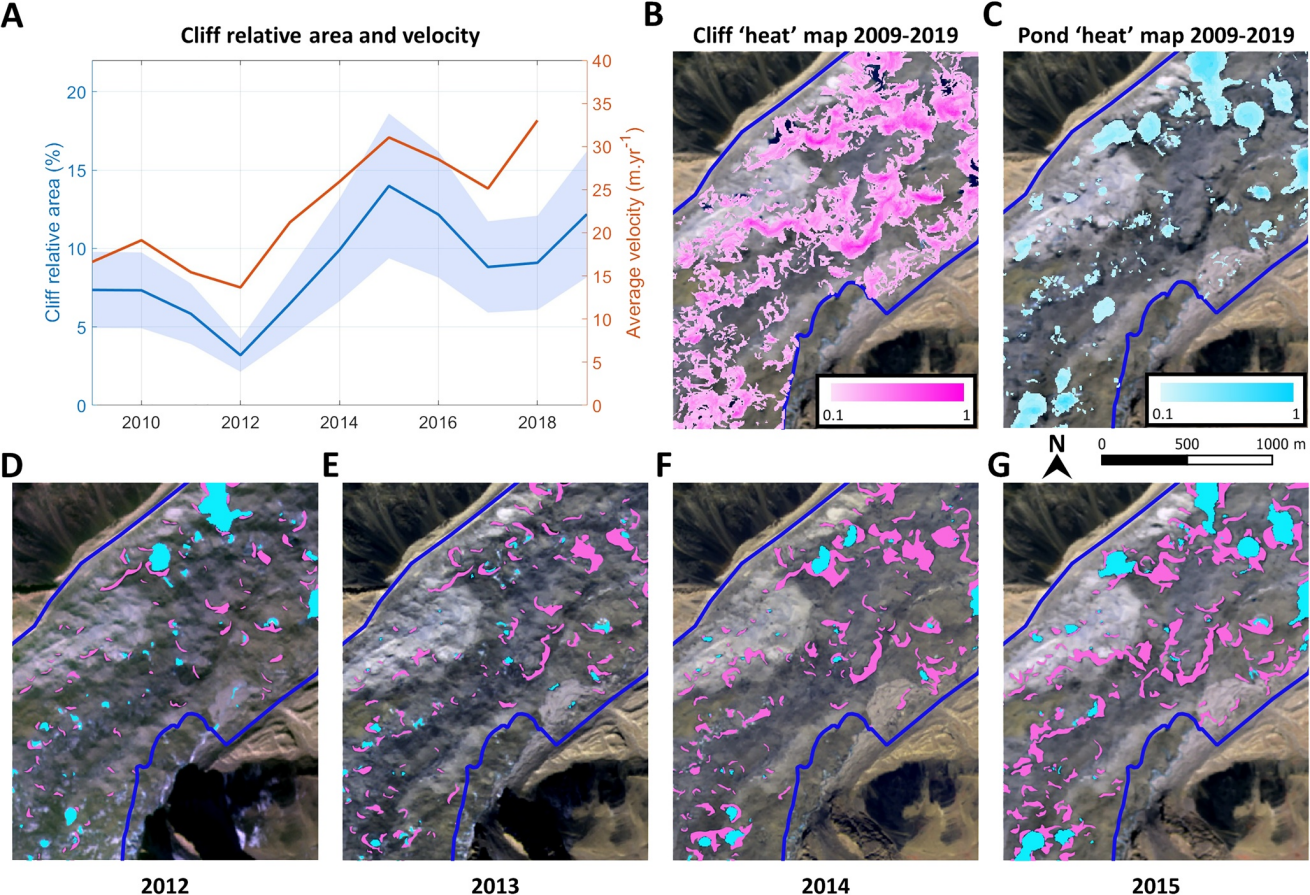
Development of cryo‐valleys between 2012 and 2015 on Urdok Glacier. (a) Cliff relative area and average surface velocity along the centerline of the debris‐covered area over time. (b) and (c) Cliff and pond density maps of the lower portion of Urdok, where 0 corresponds to no cliff or pond occurrence over the whole time series and 1 corresponds to the occurrence of cliffs or ponds in all images. Background image is the 2015 RapidEye scene (color composite of bands 5, 4, and 2). (d)–(g) Maps of cliffs (pink) and ponds (blue) on the same portion of Urdok for the 2012 to 2015 RapidEye images. Background images are the corresponding RapidEye scenes (color composite of bands 5, 4, and 2).

Urdok is an interesting study site as its cliff population undergoes extreme variations, and shows a major increase in cliff relative area between years 2012 and 2015 that the internal variability fails to explain. This change results from an increase in cliff size from merge, mix and persist events (Figure 4) and coincides with a strong increase in the average velocity of the debris‐covered area (Figure 7) that could be indicative of a surge event. The fact that this increase in cliff relative area is driven by merge and mix events is a sign that there is a large reorganization of the cliffs at the scale of the glacier, while at the same time they increase in size. The comparison of cliff outlines between 2012 and 2015 shows that the cliffs expand laterally in the direction of their principal axis along sinuous paths across the glacier surface (Figures 7d–7g). This can be interpreted as the rapid development of large “cryo‐valleys” at the surface of the glacier, as these cliffs develop and expand on either side of supraglacial streams, thus reshaping the surface of the glacier within the span of a few years. Similar mechanisms have been described on Zmutt Glacier (Mölg et al., 2020) but over the course of several decades and without the occurrence of a glacier surge. Rather, the development of cryo‐valleys at Zmutt seems to have occurred as the glacier stagnated, leading to persistent configurations of the glacier's drainage network (Mölg et al., 2020), whereas in the case of Urdok, the cryo‐valley development coincides with an up‐glacier (but not local) increase in velocity, suggesting reorganization of drainage networks driving water to the glacier's surface above the study area, which is supported by the erratic interannual fluctuations of pond area observations during this period (Figure 3). Understanding the mechanism of Urdok Glacier's increase in velocity is outside the scope of the present study and an opportunity for further investigation, but the example shows clearly that cliff and pond populations can undergo considerable changes due to external drivers.

Our stochastic model lets us quantify the possible range of the number and relative area of ice cliffs and our observations fall within the model bounds in all cases except for Urdok. There, the glacier is undergoing a major change which results in the complete rearrangement of the cliffs at its surface. We interpret the variability bounds given by the model as a physical constraint on the system. These bounds define the cliff carrying capacity of a glacier. Indeed, the number and relative area of cliffs are constrained by the availability of steep slopes, which are in turn constrained by the number of hummocks and cryo‐valleys (Bartlett et al., 2020; King et al., 2020; Mölg et al., 2020). The size of the hummocks is constrained by the glacier size (Bartlett et al., 2020), which gives an upper bound to the cliff relative area. However, the development of cryo‐valleys enables cliffs to arrange themselves in a very different and denser way than on a purely hummocky surface, and this transition is clearly visible in the case of Urdok (Figure 7). The exact reasons for this transition are unclear but are probably linked to the surge event highlighted by the change in velocity of the debris‐covered area, which impacts the glacier hydrological system (Chudley & Willis, 2019; Gulley et al., 2009; Miles, Willis, et al., 2017; Quincey et al., 2015). Indeed, the data for Urdok suggests that a surge front migrated through the upper part of the glacier (above the AOI) with little impact on the velocity or strain rates in the AOI but resulted in the routing of more water at the surface of the AOI, thus leading to the development of cryo‐valleys. Therefore, the ice cliff population is expected to evolve within the bounds given by the stochastic model parameters, but this steady state can be modified by intense changes in surface topography resulting from major glaciological or climatic changes. As a result, we expect that the state and distribution of ice cliffs on a glacier could inform us to some extent about its dynamic state and climatic drivers.

**Figure 8:**
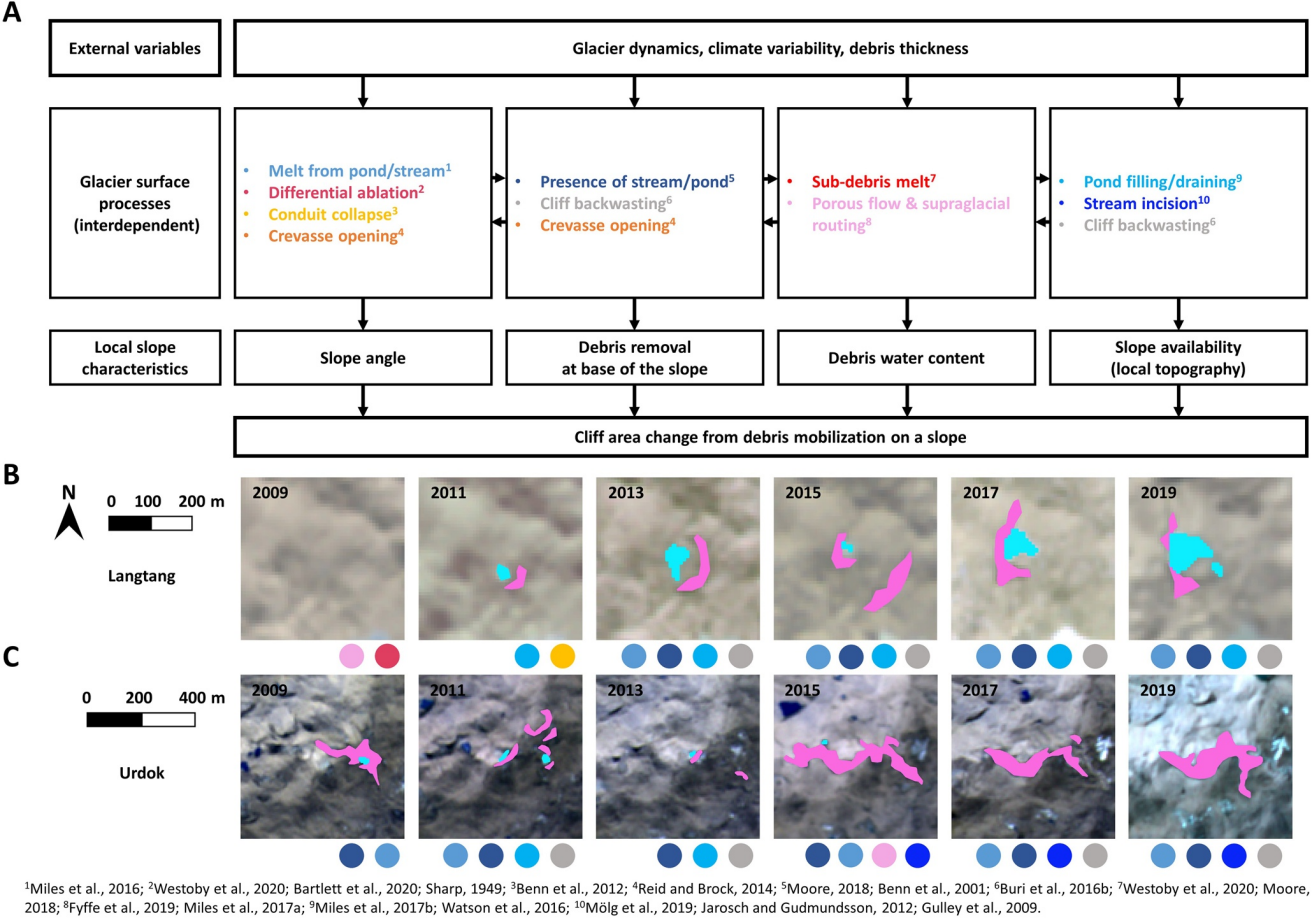
(a) Processes influencing ice cliff area change from the local slope to the glacier scale. The glacier surface processes are all attributed to a different color. (b and c) Evolution of a cliff‐pond system on Langtang Glacier and a cryo‐valley cliff on Urdok Glacier from 2009 to 2019. The pink outlines correspond to the different states of the cliffs that first appeared in the images in 2011 for Langtang and 2009 for Urdok. The associated ponds are represented in blue. The colored dots at the base of each map represent the most likely glacier surface processes at play at this location in the previous two years.

The fact that none of the tested variables stands out as a main driver of the cliff variability highlights the complexity underlying ice cliff evolution, due to a number of competing and interlinked processes happening at the glacier surface (Figure 8). Indeed, changes in glacier velocity or climate may translate differently at the local scale depending on the local hydrology, debris thickness, and topography. At this local slope scale, the cliff area change, including formation or decay, is ruled by debris mobilization, which depends on local slope characteristics (Moore, 2018). These include the slope angle, the debris water content along with the state of the base of the slope and the possibility for sliding debris to be removed (Moore, 2018), but also on the surrounding topography which may constrain the cliff's lateral expansion. A number of processes that are interdependent and difficult to quantify at larger scales may modify these slope characteristics. For example, the development of a supraglacial stream or pond from sub‐debris melt and in‐debris flow routing (Fyffe et al., 2019; Miles, Steiner, et al., 2017; Westoby et al., 2020) has the combined effect of increasing the melt at the base of the slope and removing the debris sliding down it (Benn et al., 2001; Miles et al., 2016; Moore, 2018). The development of supraglacial streams is therefore beneficial to an increase in cliff relative area along cryo‐valleys (Mölg et al., 2019) as long as the incision rate does not exceed the sub‐debris melt rate (e.g., Reid & Brock, 2010), which would lead the stream to form an englacial conduit via a cut‐and‐closure mechanism (Gulley et al., 2009; Jarosch & Gudmundsson, 2012). Such a stream could however be interrupted by the opening of a crevasse, which depends on the glacier strain rates, while at the same time such crevasses could initiate ice cliff formation via an increase of the slope angle and the removal of debris (Reid & Brock, 2014). Crevasses may also affect flow routing and therefore the draining or filling of ponds (Miles, Willis, et al., 2017; Watson et al., 2016), with consequences on melt or slope availability for cliffs (Miles et al., 2016). These processes are all influenced by external variables such as climate and glacier dynamics, but also depend on the local topography (Figure 8). When looking at the cliff population of a glacier in the long term, the stochastic approach ignores these processes that are conceptually understood or at least hypothesized, but difficult to measure. However, in some cases like for Urdok Glacier, they may trigger major changes in the cliff relative area, which calls for a need to quantify these processes more accurately to better understand the drivers of cliff evolution.

### 6.4 Outlooks

The significant variability of ice cliffs that we observed shows that cliff population dynamics need to be taken into account for distributed glacier melt models operating at multi‐year timescales. Indeed, for example on Langtang, an increase in cliff relative area of 20% could translate into 4% of additional volume loss of the debris‐covered area (Buri et al., 2021). The stochastic cliff birth‐death models are computationally efficient tools to represent this variability and an interesting next step would be to analyze backwasting rates in combination with the different events we described here. This would likely require high‐resolution DEMs for the cliff time series or at least solid assumptions for cliff slope. For this purpose, and despite available cliff energy‐balance models (Buri, Miles, et al., 2016; Juen et al., 2014), a better understanding of the relationship between the different steps of the cliff life cycle, debris redistribution, and action of supraglacial streams and ponds, especially during the melt season, would be very valuable. It has been suggested that cliffs could increase in size during the monsoon season (Steiner et al., 2019) due to debris mobilization from precipitation and melt, but such a seasonality has not been clearly observed; a more detailed analysis of what happens to cliffs sub‐seasonally, and especially during the monsoon, and the implications for cliff‐associated melt would thus be highly relevant. A better understanding of these processes would also help improve the stochastic model that is, at present, highly empirical and glacier‐specific. Quantifying various surface processes, understanding their spatial and temporal scales, and linking them with climatic variables and glacier dynamics will likely enable a more robust representation of ice cliff variability.

This study also shows that there can be very different ice cliff population dynamics for different glaciers, which depend at least partially on the mechanisms that drive cliff birth and organization. However, this variability is difficult to constrain due to the lack of large‐scale observations of ice cliffs. The emergence of semi‐automated approaches to map these features from remote sensing data (Anderson et al., 2021; Herreid & Pellicciotti, 2018; Kneib et al., 2020) should enable this large‐scale mapping and as a result a better understanding of cliffs' broad spatial variability. Finally, the cliff tracking approach and data set along with the model developed here offer an opportunity to further investigate the causes of individual and bulk cliff behavior and thus better understand the local drivers of cliff birth and death and the possible influence of the local topography, surface hydrology, geomorphology or glacier motion.

## 7 Conclusions

In this study, we combined systematic mapping of ice cliffs at yearly intervals with a method to track individual cliffs to quantify and characterize the cliff population variability of four HMA glaciers. Our results show that the cliff relative area can commonly change by 20% and up to 80% from one year to the next at the surface of a glacier. Due to the melt enhancement effect of the cliffs, this variability will have large implications for the melt of debris‐covered glaciers and should therefore be accounted for in glacier and glacio‐hydrological models, at least in a stochastic way in a first step to include this additional uncertainty from natural cliff variability in the melt rates.

This interannual variability is driven by a combination of contributions from different events occurring at the cliff scale and that rule the cliff life cycle. Birth and death events dominate the variability in the number of ice cliffs. Death events constrain the number of ice cliffs, thus defining the cliff carrying capacity of a glacier while birth events are stochastic, with some dependence on the pond interannual variability. These ponds promote cliff persistence but affect less than 50% of the population and thus are not the main driver of the cliff population's interannual variability. The changes in cliff relative area are also driven by the net contributions of persist events in addition to net contributions from split, merge, or mix events that vary consistently.

These events can be represented in a stochastic birth‐death model to constrain the long‐term natural variability of the number and area of cliffs. Some of the changes are however not entirely stochastic and our results show that they can be influenced by climate, supraglacial ponds and/or surface velocity, in spite of the complexity of all the interdependent processes occurring at the glacier surface. As a result, major climatic or glaciological changes, such as we have seen in the case of a surge, may lead to a reorganization of ice cliffs at the glacier surface and a change in the natural variability of the system.

## Supporting information

Supporting Information S1

## Data Availability

All the cliff and pond outlines generated for this study are available on Zenodo (https://doi.org/10.5281/zenodo.4632840) along with the different codes used, including the ones used for the tracking and the modeling (Kneib et al., 2021). Other data sets used for this research are elevation changes (Brun et al., 2017a), surface velocity data from ITS_LIVE (Dehecq et al., 2019; Gardner et al., 2018), climate data from ERA5‐Land (Muñoz Sabater, 2019), and debris‐covered glacier outlines (Herreid & Pellicciotti, 2020a).

## References

[jgrf21431-bib-0001] Altena, B. , & Kääb, A. (2020). Satellite remote sensing of ice cliff migration. Retrieved from https://ui.adsabs.harvard.edu/abs/2020EGUGA.22.5057A/abstract

[jgrf21431-bib-0002] Anderson, L. S. , & Anderson, R. S. (2016). Modeling debris‐covered glaciers: Response to steady debris deposition. The Cryosphere, 10, 1105–1124. 10.5194/tc-10-1105-2016

[jgrf21431-bib-0003] Anderson, L. S. , & Anderson, R. S. (2018). Debris thickness patterns on debris‐covered glaciers. Geomorphology, 311, 1–12. 10.1016/j.geomorph.2018.03.014

[jgrf21431-bib-0004] Anderson, L. S. , Armstrong, W. H. , Anderson, R. S. , & Buri, P. (2021). Debris cover and the thinning of Kennicott Glacier, Alaska: In situ measurements, automated ice cliff delineation and distributed melt estimates. The Cryosphere, 15(1), 265–282. 10.5194/tc-15-265-2021

[jgrf21431-bib-0005] Bailey, N. T. (1968). Stochastic birth, death and migration processes for spatially distributed populations. Biometrika, 55(1), 189–198. 10.1093/biomet/55.1.189 5661044

[jgrf21431-bib-0006] Bartlett, O. T. , Ng, F. S. L. , & Rowan, A. V. (2020). Morphology and evolution of supraglacial hummocks on debris‐covered Himalayan glaciers. Earth Surface Processes and Landforms, 5043. 10.1002/esp.5043

[jgrf21431-bib-0007] Benn, D. I. , Bolch, T. , Hands, K. , Gulley, J. , Luckman, A. , Nicholson, L. I. , et al. (2012). Response of debris‐covered glaciers in the Mount Everest region to recent warming, and implications for outburst flood hazards. Earth‐Science Reviews, 114, 156–174. 10.1016/j.earscirev.2012.03.008

[jgrf21431-bib-0008] Benn, D. I. , Wiseman, S. , & Hands, K. A. (2001). Growth and drainage of supraglacial lakes on debris mantled Ngozumpa Glacier, Khumbu Himal, Nepal. Journal of Glaciology, 47(159), 626–638. 10.3189/172756501781831729

[jgrf21431-bib-0009] Bhambri, R. , Hewitt, K. , Kawishwar, P. , & Pratap, B. (2017). Surge‐type and surge‐modified glaciers in the Karakoram. Scientific Reports, 7(1), 1–14. 10.1038/s41598-017-15473-8 29133812 PMC5684366

[jgrf21431-bib-0010] Brun, F. , Berthier, E. , Wagnon, P. , Kääb, A. , & Treichler, D. (2017a). Elevation changes of High Mountain Asia from 2000 to 2016, links to GeoTIFFs. PANGAEA. 10.1594/PANGAEA.876545

[jgrf21431-bib-0011] Brun, F. , Berthier, E. , Wagnon, P. , Kääb, A. , & Treichler, D. (2017b). A spatially resolved estimate of High Mountain Asia glacier mass balances from 2000 to 2016. Nature Geoscience, 10, 668–673. 10.1038/ngeo2999 PMC5584675 28890734

[jgrf21431-bib-0012] Brun, F. , Buri, P. , Miles, E. S. , Wagnon, P. , Steiner, J. , Berthier, E. , et al. (2016). Quantifying volume loss from ice cliffs on debris‐covered glaciers using high‐resolution terrestrial and aerial photogrammetry. Journal of Glaciology, 62, 684–695. 10.1017/jog.2016.54

[jgrf21431-bib-0013] Brun, F. , Wagnon, P. , Berthier, E. , Shea, J. M. , Immerzeel, W. W. , Kraaijenbrink, P. D. A. , et al. (2018). Ice cliff contribution to the tongue‐wide ablation of Changri Nup Glacier, Nepal, central Himalaya. The Cryosphere, 12(11), 3439–3457. 10.5194/tc-12-3439-2018

[jgrf21431-bib-0014] Buri, P. , Miles, E. S. , Steiner, J. F. , Immerzeel, W. W. , Wagnon, P. , & Pellicciotti, F. (2016). A physically based 3‐D model of ice cliff evolution over debris‐covered glaciers. Journal of Geophysical Research: Earth Surface, 121(12), 2471–2493. 10.1002/2016JF004039

[jgrf21431-bib-0015] Buri, P. , Miles, E. S. , Steiner, J. F. , Ragettli, S. , & Pellicciotti, F. (2021). Supraglacial ice cliffs can substantially increase the mass loss of debris‐covered glaciers. Geophysical Research Letters, 48(6). 10.1029/2020GL092150

[jgrf21431-bib-0016] Buri, P. , & Pellicciotti, F. (2018). Aspect controls the survival of ice cliffs on debris‐covered glaciers. Proceedings of the National Academy of Sciences of the United States of America, 115(17), 4369–4374. 10.1073/pnas.1713892115 29632176 PMC5924879

[jgrf21431-bib-0017] Buri, P. , Pellicciotti, F. , Steiner, J. F. , Miles, E. S. , & Immerzeel, W. W. (2016). A grid‐based model of backwasting of supraglacial ice cliffs on debris‐covered glaciers. Annals of Glaciology, 57(71), 199–211. 10.3189/2016AoG71A059

[jgrf21431-bib-0018] Chinn, T. J. H. , & Dillon, A. (1987). Observations on a debris‐covered polar glacier “Whisky Glacier,” James Ross Island, Antarctic Peninsula, Antarctica. Journal of Glaciology, 33(115), 300–310. 10.3189/s002214300000887x

[jgrf21431-bib-0019] Chudley, T. R. , & Willis, I. C. (2019). Glacier surges in the north‐west West Kunlun Shan inferred from 1972 to 2017 Landsat imagery. Journal of Glaciology, 65(249), 1–12. 10.1017/jog.2018.94

[jgrf21431-bib-0020] Dehecq, A. , Gourmelen, N. , Gardner, A. S. , Brun, F. , Goldberg, D. , Nienow, P. W. , et al. (2019). Twenty‐first century glacier slowdown driven by mass loss in High Mountain Asia. Nature Geoscience, 12(1), 22–27. 10.1038/s41561-018-0271-9

[jgrf21431-bib-0021] Ferguson, J. , & Vieli, A. (2021). Modelling steady states and the transient response of debris‐covered glaciers. The Cryosphere. 15(7), 3377–3399. 10.5194/tc-2020-228

[jgrf21431-bib-0022] Fyffe, C. L. , Brock, B. W. , Kirkbride, M. P. , Mair, D. W. F. , Arnold, N. S. , Smiraglia, C. , et al. (2019). Do debris‐covered glaciers demonstrate distinctive hydrological behaviour compared to clean glaciers? Journal of Hydrology, 570, 584–597. 10.1016/j.jhydrol.2018.12.069

[jgrf21431-bib-0023] Gardelle, J. , Berthier, E. , Arnaud, Y. , & Kääb, A. (2013). Region‐wide glacier mass balances over the Pamir‐Karakoram‐Himalaya during 1999–2011. The Cryosphere, 7, 1263–1286. 10.5194/tc-7-1263-2013

[jgrf21431-bib-0024] Gardner, A. S. , Moholdt, G. , Scambos, T. , Fahnstock, M. , Ligtenberg, S. , Van Den Broeke, M. , & Nilsson, J. (2018). Increased West Antarctic and unchanged East Antarctic ice discharge over the last 7 years. The Cryosphere, 12(2), 521–547. 10.5194/tc-12-521-2018

[jgrf21431-bib-0025] Gulley, J. D. , Benn, D. I. , Screaton, E. , & Martin, J. (2009). Mechanisms of englacial conduit formation and their implications for subglacial recharge. Quaternary Science Reviews, 28(19–20), 1984–1999. 10.1016/j.quascirev.2009.04.002

[jgrf21431-bib-0026] Hagg, W. , Mayr, E. , Mannig, B. , Reyers, M. , Schubert, D. , Pinto, J. , et al. (2018). Future climate change and its impact on runoff generation from the debris‐covered Inylchek Glaciers, Central Tian Shan, Kyrgyzstan. Water, 10(11), 1513. 10.3390/w10111513

[jgrf21431-bib-0027] Han, H. , Wang, J. , Wei, J. , & Liu, S. (2010). Backwasting rate on debris‐covered Koxkar glacier, Tuomuer Mountain, China. Journal of Glaciology, 56(19). 10.3189/002214310791968430

[jgrf21431-bib-0028] Herreid, S. , & Pellicciotti, F. (2018). Automated detection of ice cliffs within supraglacial debris cover. The Cryosphere, 12, 1811–1829. 10.5194/tc-12-1811-2018

[jgrf21431-bib-0029] Herreid, S. , & Pellicciotti, F. (2020a). Supplementary Information for Herreid and Pellicciotti, Nature Geoscience, 2020 (Version 1.0). Nature Geoscience. 10.5281/zenodo.3866466

[jgrf21431-bib-0030] Herreid, S. , & Pellicciotti, F. (2020b). The state of rock debris covering Earth's glaciers. Nature Geoscience, 13, 1–627. 10.1038/s41561-020-0615-0

[jgrf21431-bib-0031] Huggel, C. , Kääb, A. , Haeberli, W. , Teysseire, P. , & Paul, F. (2002). Remote sensing based assessment of hazards from glacier lake outbursts: A case study in the Swiss Alps. Canadian Geotechnical Journal, 39(2), 316–330. 10.1139/t01-099

[jgrf21431-bib-0032] Huss, M. (2013). Density assumptions for converting geodetic glacier volume change to mass change. The Cryosphere, 7(3), 877–887. 10.5194/tc-7-877-2013

[jgrf21431-bib-0033] Immerzeel, W. W. , Kraaijenbrink, P. D. A. , Shea, J. M. , Shrestha, A. B. , Pellicciotti, F. , Bierkens, M. F. P. , & De Jong, S. M. (2014). High‐resolution monitoring of Himalayan glacier dynamics using unmanned aerial vehicles. Remote Sensing of Environment, 150, 93–103. 10.1016/j.rse.2014.04.025

[jgrf21431-bib-0034] Inoue, J. , & Yoshida, M. (1980). Ablation and heat exchange over the Khumbu glacier ablation and heat exchange over the Khumbu glacier. Journal of the Japanese Society of Snow and Ice, 41(Special), 26–33.

[jgrf21431-bib-0035] Jarosch, A. H. , & Gudmundsson, M. T. (2012). A numerical model for meltwater channel evolution in glaciers. The Cryosphere, 6(2), 493–503. 10.5194/tc-6-493-2012

[jgrf21431-bib-0036] Johnson, P. G. (1992). Stagnant glacier ice, St. Elias Mountains, Yukon. Geografiska Annaler—Series A: Physical Geography, 74(1), 13–19. 10.2307/521466

[jgrf21431-bib-0037] Juen, M. , Mayer, C. , Lambrecht, A. , Han, H. , & Liu, S. (2014). Impact of varying debris cover thickness on ablation: A case study for Koxkar Glacier in the Tien Shan. The Cryosphere, 8(2), 377–386. 10.5194/tc-8-377-2014

[jgrf21431-bib-0038] Kääb, A. , Berthier, E. , Nuth, C. , Gardelle, J. , & Arnaud, Y. (2012). Contrasting patterns of early twenty‐first‐century glacier mass change in the Himalayas. Nature, 488, 495–498. 10.1038/nature11324 22914167

[jgrf21431-bib-0039] Kargel, J. S. , Leonard, G. J. , Shugar, D. H. , Haritashya, U. K. , Bevington, A. , Fielding, E. J. , et al. (2016). Geomorphic and geologic controls of geohazards induced by Nepal's 2015 Gorkha earthquake. Science, 351(6269). 10.1126/science.aac8353 26676355

[jgrf21431-bib-0040] Kendall, D. G. (1948). On the generalized “birth‐and‐death” process. The Annals of Mathematical Statistics, 19(1), 1–15. 10.1214/aoms/1177730285

[jgrf21431-bib-0041] King, O. , Turner, A. G. D. , Quincey, D. J. , & Carrivick, J. L. (2020). Morphometric evolution of Everest region debris‐covered glaciers. Geomorphology, 371, 107422. 10.1016/j.geomorph.2020.107422

[jgrf21431-bib-0043] Kneib, M. , Miles, E. S. , Buri, P. , Molnar, P. , McCarthy, M. , Fugger, S. , & Pellicciotti, F. (2021). Data and scripts for interannual dynamics of ice cliff populations on debris‐covered glaciers from remote sensing observations and stochastic modeling. Zenodo. 10.5281/zenodo.4632840 PMC9285626 35860443

[jgrf21431-bib-0042] Kneib, M. , Miles, E. S. , Jola, S. , Buri, P. , Herreid, S. , Bhattacharya, A. , et al. (2020). Mapping ice cliffs on debris‐covered glaciers using multispectral satellite images. Remote Sensing of Environment, 253, 112201. 10.1016/j.rse.2020.112201

[jgrf21431-bib-0045] Kraaijenbrink, P. D. A. , Bierkens, M. F. P. , Lutz, A. F. , & Immerzeel, W. W. (2017). Impact of a global temperature rise of 1.5 degrees Celsius on Asia's glaciers. Nature, 549, 257–260. 10.1038/nature23878 28905897

[jgrf21431-bib-0044] Kraaijenbrink, P. D. A. , Meijer, S. W. , Shea, J. M. , Pellicciotti, F. , De Jong, S. M. , & Immerzeel, W. W. (2016). Seasonal surface velocities of a Himalayan glacier derived by automated correlation of unmanned aerial vehicle imagery. Annals of Glaciology, 57(71), 103–113. 10.3189/2016AoG71A072

[jgrf21431-bib-0046] Kraaijenbrink, P. D. A. , Shea, J. M. , Pellicciotti, F. , De Jong, S. M. , & Immerzeel, W. W. (2016). Object‐based analysis of unmanned aerial vehicle imagery to map and characterise surface features on a debris‐covered glacier. Remote Sensing of Environment, 186, 581–595. 10.1016/j.rse.2016.09.013

[jgrf21431-bib-0047] McFeeters, S. K. (1996). The use of the Normalized Difference Water Index (NDWI) in the delineation of open water features. International Journal of Remote Sensing, 17(7), 1425–1432. 10.1080/01431169608948714

[jgrf21431-bib-0048] Messerli, A. , & Grinsted, A. (2015). Image georectification and feature tracking toolbox: ImGRAFT. Geoscientific Instrumentation, Methods and Data Systems, 4(1), 23–34. 10.5194/gi-4-23-2015

[jgrf21431-bib-0049] Miles, E. S. , Pellicciotti, F. , Willis, I. C. , Steiner, J. F. , Buri, P. , & Arnold, N. S. (2016). Refined energy‐balance modelling of a supraglacial pond, Langtang Khola, Nepal. Annals of Glaciology, 57, 29–40. 10.3189/2016AoG71A421

[jgrf21431-bib-0050] Miles, E. S. , Steiner, J. , Willis, I. , Buri, P. , Immerzeel, W. W. , Chesnokova, A. , & Pellicciotti, F. (2017). Pond dynamics and supraglacial‐englacial connectivity on debris‐covered Lirung Glacier, Nepal. Frontiers of Earth Science, 5. 10.3389/feart.2017.00069

[jgrf21431-bib-0051] Miles, E. S. , Willis, I. , Buri, P. , Steiner, J. F. , Arnold, N. S. , & Pellicciotti, F. (2018). Surface pond energy absorption across four Himalayan glaciers accounts for 1/8 of total catchment ice loss. Geophysical Research Letters, 45(19), 10464–10473. 10.1029/2018GL079678 31031450 PMC6473701

[jgrf21431-bib-0052] Miles, E. S. , Willis, I. C. , Arnold, N. S. , Steiner, J. , & Pellicciotti, F. (2017). Spatial, seasonal and interannual variability of supraglacial ponds in the Langtang Valley of Nepal. Journal of Glaciology, 63(237), 88–105. 10.1017/jog.2016.120

[jgrf21431-bib-0053] Miles, J. (2014). R squared, adjusted R squared. In Wiley StatsRef: Statistics reference online. John Wiley & Sons, Ltd. 10.1002/9781118445112.stat06627

[jgrf21431-bib-0054] Mölg, N. , Bolch, T. , Walter, A. , & Vieli, A. (2019). Unravelling the evolution of Zmuttgletscher and its debris cover since the end of the Little Ice Age. The Cryosphere, 13(7), 1889–1909. 10.5194/tc-13-1889-2019

[jgrf21431-bib-0055] Mölg, N. , Ferguson, J. , Bolch, T. , & Vieli, A. (2020). On the influence of debris cover on glacier morphology: How high‐relief structures evolve from smooth surfaces. Geomorphology, 357, 107092. 10.1016/j.geomorph.2020.107092

[jgrf21431-bib-0056] Moore, P. L. (2018). Stability of supraglacial debris. Earth Surface Processes and Landforms, 43(1), 285–297. 10.1002/esp.4244

[jgrf21431-bib-0057] Muñoz Sabater, J. (2019). ERA5‐Land monthly averaged data from 1981 to present. Copernicus Climate Change Service (C3S) Climate Data Store (CDS). 10.24381/cds.68d2bb30

[jgrf21431-bib-0058] Nainwal, H. C. , Banerjee, A. , Shankar, R. , Semwal, P. , & Sharma, T. (2016). Shrinkage of Satopanth and Bhagirath Kharak Glaciers, India, from 1936 to 2013. Annals of Glaciology, 57(71), 131–139. 10.3189/2016aog71a015

[jgrf21431-bib-0059] Nicholson, L. I. , McCarthy, M. , Pritchard, H. D. , & Willis, I. (2018). Supraglacial debris thickness variability: Impact on ablation and relation to terrain properties. The Cryosphere, 12(12), 3719–3734. 10.5194/tc-12-3719-2018

[jgrf21431-bib-0060] Ogilvie, I. H. (1904). The effect of superglacial débris on the advance and retreat of some Canadian glaciers. The Journal of Geology, 12(8), 722–743. 10.1086/621194

[jgrf21431-bib-0061] Ostrem, G. (1959). Ice melting under a thin layer of Moraine, and the existence of ice cores in Moraine Ridges. Geografiska Annaler, 41(4), 228–230. 10.1080/20014422.1959.11907953

[jgrf21431-bib-0062] Paschalis, A. , Molnar, P. , Fatichi, S. , & Burlando, P. (2013). A stochastic model for high‐resolution space‐time precipitation simulation. Water Resources Research, 49(12), 8400–8417. 10.1002/2013WR014437

[jgrf21431-bib-0063] Pellicciotti, F. , Stephan, C. , Miles, E. S. , Herreid, S. , Immerzeel, W. W. , & Bolch, T. (2015). Mass‐balance changes of the debris‐covered glaciers in the Langtang Himal, Nepal, from 1974 to 1999. Journal of Glaciology, 61(226), 373–386. 10.3189/2015JoG13J237

[jgrf21431-bib-0064] Planet Team . (2017). Planet application program interface. In Space for life on Earth. Retrieved from https://api.planet.com

[jgrf21431-bib-0065] Quincey, D. J. , Glasser, N. F. , Cook, S. J. , & Luckman, A. (2015). Heterogeneity in Karakoram glacier surges. Journal of Geophysical Research: Earth Surface, 120(7), 1288–1300. 10.1002/2015JF003515

[jgrf21431-bib-0066] Ragettli, S. , Bolch, T. , & Pellicciotti, F. (2016). Heterogeneous glacier thinning patterns over the last 40 years in Langtang Himal, Nepal. The Cryosphere, 10, 2075–2097. 10.5194/tc-10-2075-2016

[jgrf21431-bib-0067] Ragettli, S. , Pellicciotti, F. , Immerzeel, W. W. , Miles, E. S. , Petersen, L. , Heynen, M. , et al. (2015). Unraveling the hydrology of a Himalayan catchment through integration of high resolution in situ data and remote sensing with an advanced simulation model. Advances in Water Resources, 78, 94–111. 10.1016/j.advwatres.2015.01.013

[jgrf21431-bib-0068] Reid, T. D. , & Brock, B. W. (2010). An energy‐balance model for debris‐covered glaciers including heat conduction through the debris layer. Journal of Glaciology, 56(199). 10.3189/002214310794457218

[jgrf21431-bib-0069] Reid, T. D. , & Brock, B. W. (2014). Assessing ice‐cliff backwasting and its contribution to total ablation of debris‐covered Miage glacier, Mont Blanc massif, Italy. Journal of Glaciology, 60(219). 10.3189/2014JoG13J045

[jgrf21431-bib-0070] RGI Consortium . (2017). Randolph glacier inventory—A dataset of global glacier outlines: Version 6.0: Technical report, global land ice measurements from space. Digital Media. 10.7265/N5-RGI-60

[jgrf21431-bib-0071] Röhl, K. (2006). Thermo‐erosional notch development at fresh‐water‐calving Tasman Glacier, New Zealand. Journal of Glaciology, 52(177), 203–213. 10.3189/172756506781828773

[jgrf21431-bib-0072] Röhl, K. (2008). Characteristics and evolution of supraglacial ponds on debris‐covered Tasman Glacier, New Zealand. Journal of Glaciology. 54(188), 867–880. 10.3189/002214308787779861

[jgrf21431-bib-0073] Rowan, A. V. , Egholm, D. L. , Quincey, D. J. , & Glasser, N. F. (2015). Modelling the feedbacks between mass balance, ice flow and debris transport to predict the response to climate change of debris‐covered glaciers in the Himalaya. Earth and Planetary Science Letters, 430, 427–438. 10.1016/j.epsl.2015.09.004

[jgrf21431-bib-0074] Sakai, A. , Nakawo, M. , & Fujita, K. (1998). Melt rate of ice cliffs on the Lirung Glacier, Nepal Himalayas, 1996. Bulletin of Glacier Research, 16, 57–66.

[jgrf21431-bib-0075] Sakai, A. , Nakawo, M. , & Fujita, K. (2002). Distribution characteristics and energy balance of ice cliffs on debris‐covered glaciers, Nepal Himalaya. Arctic Antarctic, and Alpine Research, 34(1), 12–19. 10.1080/15230430.2002.12003463

[jgrf21431-bib-0076] Sakai, A. , & Takeuchi, N. (2000). Debris‐covered glaciers. IAHS Publication.

[jgrf21431-bib-0077] Salerno, F. , Thakuri, S. , D'Agata, C. , Smiraglia, C. , Manfredi, E. C. , Viviano, G. , & Tartari, G. (2012). Glacial lake distribution in the Mount Everest region: Uncertainty of measurement and conditions of formation. Global and Planetary Change, 92–93, 30–39. 10.1016/j.gloplacha.2012.04.001

[jgrf21431-bib-0078] Scherler, D. , Wulf, H. , & Gorelick, N. (2018). Global assessment of supraglacial debris‐cover extents. Geophysical Research Letters, 45, 11,798–11,805. 10.1029/2018GL080158

[jgrf21431-bib-0079] Shah, S. S. , Banerjee, A. , Nainwal, H. C. , & Shankar, R. (2019). Estimation of the total sub‐debris ablation from point‐scale ablation data on a debris‐covered glacier. Journal of Glaciology, 65(253), 759–769. 10.1017/jog.2019.48

[jgrf21431-bib-0080] Shahgedanova, M. , Stokes, C. R. , Gurney, S. D. , & Popovnin, V. (2005). Interactions between mass balance, atmospheric circulation, and recent climate change on the Djankuat Glacier, Caucasus Mountains, Russia. Journal of Geophysical Research: Atmospheres, 110(4), 1–14. 10.1029/2004JD005213

[jgrf21431-bib-0081] Sharp, R. P. (1949). Studies of superglacial debris on valley glaciers. American Journal of Science, 247(5), 289–315. 10.2475/ajs.247.5.289

[jgrf21431-bib-0082] Steiner, J. F. , Buri, P. , Miles, E. S. , Ragettli, S. , & Pellicciotti, F. (2019). Supraglacial ice cliffs and ponds on debris‐covered glaciers: Spatio‐temporal distribution and characteristics. Journal of Glaciology, 65(252), 617–632. 10.1017/jog.2019.40

[jgrf21431-bib-0083] Steiner, J. F. , Pellicciotti, F. , Buri, P. , Miles, E. S. , Immerzeel, W. W. , & Reid, T. D. (2015). Modelling ice‐cliff backwasting on a debris‐covered glacier in the Nepalese Himalaya. Journal of Glaciology, 61(229), 889–907. 10.3189/2015JoG14J194

[jgrf21431-bib-0084] Thompson, S. , Benn, D. I. , Mertes, J. , & Luckman, A. (2016). Stagnation and mass loss on a Himalayan debris‐covered glacier: Processes, patterns and rates. Journal of Glaciology, 62, 467–485. 10.1017/jog.2016.37

[jgrf21431-bib-0085] Umbach, D. , & Jones, K. N. (2000). A few methods for fitting circles to data. IEEE Transactions on Instrumentation and Measurement, 52(6).

[jgrf21431-bib-0086] Watson, C. S. , King, O. , Miles, E. S. , & Quincey, D. J. (2018). Optimising NDWI supraglacial pond classification on Himalayan debris‐covered glaciers. Remote Sensing of Environment, 217, 414–425. 10.1016/j.rse.2018.08.020

[jgrf21431-bib-0087] Watson, C. S. , Quincey, D. J. , Carrivick, J. L. , & Smith, M. W. (2016). The dynamics of supraglacial ponds in the Everest region, central Himalaya. Global and Planetary Change, 142, 14–27. 10.1016/j.gloplacha.2016.04.008

[jgrf21431-bib-0088] Watson, C. S. , Quincey, D. J. , Carrivick, J. L. , & Smith, M. W. (2017). Ice cliff dynamics in the Everest region of the Central Himalaya. Geomorphology, 278, 238–251. 10.1016/j.geomorph.2016.11.017

[jgrf21431-bib-0089] Watson, C. S. , Quincey, D. J. , Smith, M. W. , Carrivick, J. L. , Rowan, A. V. , & James, M. R. (2017). Quantifying ice cliff evolution with multi‐temporal point clouds on the debris‐covered Khumbu Glacier, Nepal. Journal of Glaciology, 63, 823–837. 10.1017/jog.2017.47

[jgrf21431-bib-0090] Westoby, M. J. , Rounce, D. R. , Shaw, T. E. , Fyffe, C. L. , Moore, P. L. , Stewart, R. L. , & Brock, B. W. (2020). Geomorphological evolution of a debris‐covered glacier surface. Earth Surface Processes and Landforms, 45(14), 3431–3448. 10.1002/esp.4973

[jgrf21431-bib-0091] Wijngaard, R. R. , Steiner, J. F. , Kraaijenbrink, P. D. A. , Klug, C. , Adhikari, S. , Banerjee, A. , et al. (2019). Modeling the response of the langtang glacier and the hintereisferner to a changing climate since the little ice age. Frontiers in Earth Science, 7, 143. 10.3389/feart.2019.00143

[jgrf21431-bib-0092] Yang, W. , Zhao, C. , Westoby, M. , Yao, T. , Wang, Y. , Pellicciotti, F. , et al. (2020). Seasonal dynamics of a temperate Tibetan Glacier revealed by high‐resolution UAV photogrammetry and in situ measurements. Remote Sensing, 12(15), 2389. 10.3390/rs12152389

