# Supplementary material for: Interannual Dynamics of Ice Cliff Populations on Debris‐Covered Glaciers From Remote Sensing Observations and Stochastic Modeling
Source: J Geophys Res Earth Surf. 2021 Oct 13;126(10):e2021JF006179. doi: 10.1029/2021JF006179 (PMC9285626; doi:10.1029/2021JF006179)
Supplement: Supplementary file 1 — Supporting Information S1 [file JGRF-126-0-s001.pdf]

Journal of Geophysical Research: Earth Surface

Supporting Information for

Interannual Dynamics of Ice Cliff Populations on Debris-Covered Glaciers from Remote Sensing Observations and Stochastic Modeling

M. Kneib[1,2], E. S. Miles[1], P. Buri[1], P. Molnar[2], M. McCarthy[1], S. Fugger[1,2], and F. Pellicciotti[1,3]

[1]High Mountain Glaciers and Hydrology (HIMAL), Swiss Federal Institute for Forest, Snow and Landscape Research WSL, 8903, Birmensdorf, Switzerland
[2]Institute of Environmental Engineering, ETH Zurich, 8093 Zurich, Switzerland
[3]Department of Geography, Northumbria University, Newcastle, NE1 7RU, UK

Contents of this file

Stochastic model description
Figures S1 to S14
Tables S1 to S8

Site	Year	Sensor	Date of acquisition
Langtang	2009	RapidEye	03/11/2009
	2010	RapidEye	26/10/2010
	2011	RapidEye	16/12/2011
	2012	RapidEye	08/11/2012
	2013	RapidEye	11/10/2013
	2014	RapidEye	11/10/2014
	2015	RapidEye	25/10/2015
	2016	RapidEye	21/10/2016
	2017	RapidEye	12/11/2017
	2018	RapidEye	20/10/2018
	2019	RapidEye	14/10/2019
Urdok	2009	RapidEye	26/09/2009
	2010	RapidEye	20/11/2010
	2011	RapidEye	29/09/2011
	2012	RapidEye	27/10/2012
	2013	RapidEye	29/09/2013
	2014	RapidEye	18/08/2014
	2015	RapidEye	09/08/2015
	2016	RapidEye	30/12/2016
	2017	RapidEye	24/09/2017
	2018	RapidEye	12/09/2018

	2019	PlanetScope	04/09/2019
Satopanth & Bhagirath Kharak	2010	RapidEye	17/10/2010
	2011	RapidEye	21/09/2011
	2012	RapidEye	23/09/2012
	2013	RapidEye	20/09/2013
	2014	RapidEye	06/10/2014
	2015	RapidEye	16/09/2015
	2016	RapidEye	23/10/2016
	2017	RapidEye	02/10/2017
	2018	RapidEye	30/10/2018
	2019	PlanetScope	10/10/2019

Table S1: Multispectral satellite data used to map cliffs and ponds.

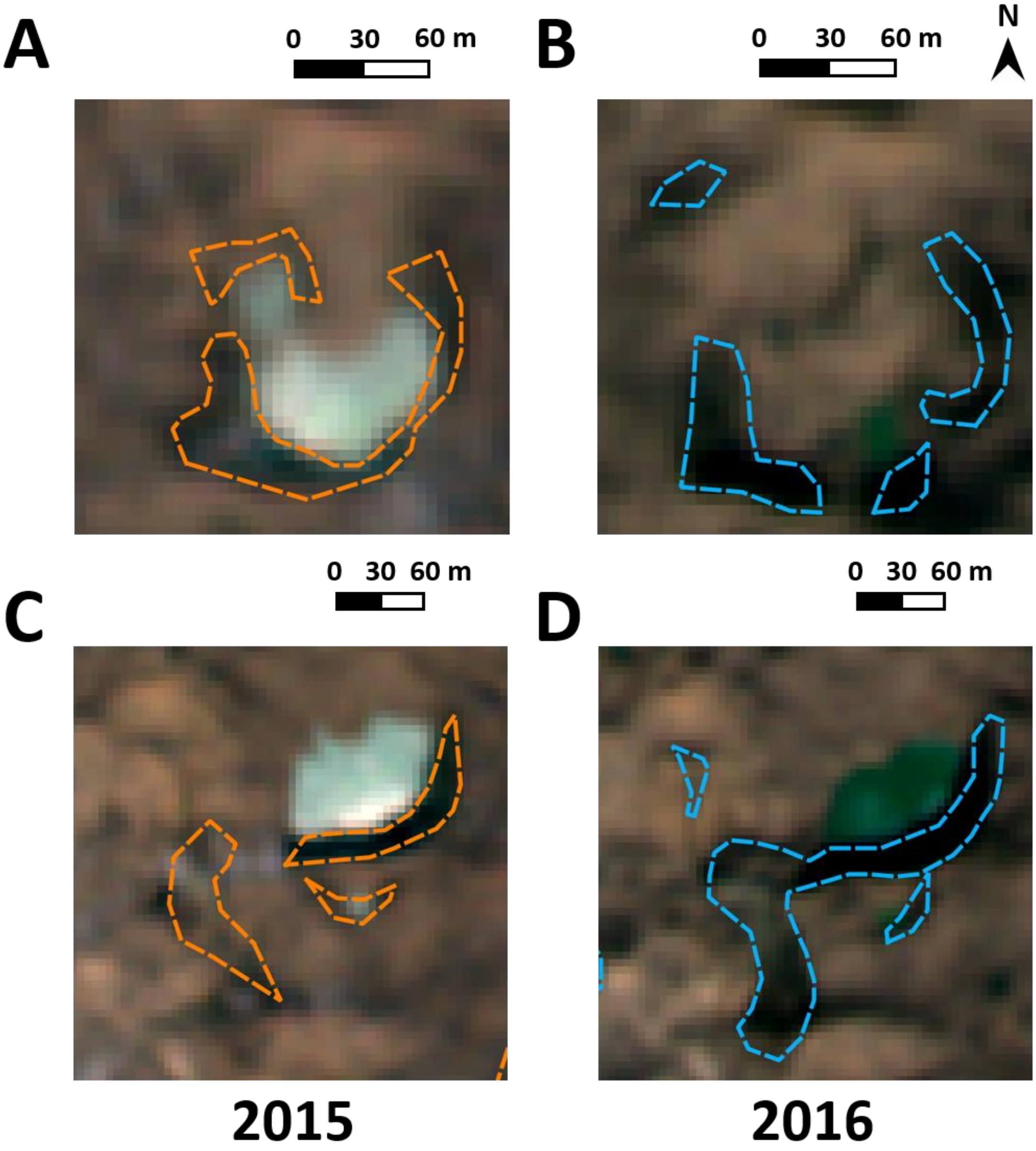

Figure S1: Mapping of ice cliffs at two locations (upper and lower panels) on Langtang Glacier from the 2015 and 2016 RapidEye images. In orange are the cliff outlines in 2015 and in blue the outlines in 2016. Background images are the RapidEye 2015 and 2016 scenes (color composite of bands 4, 2 and 1).

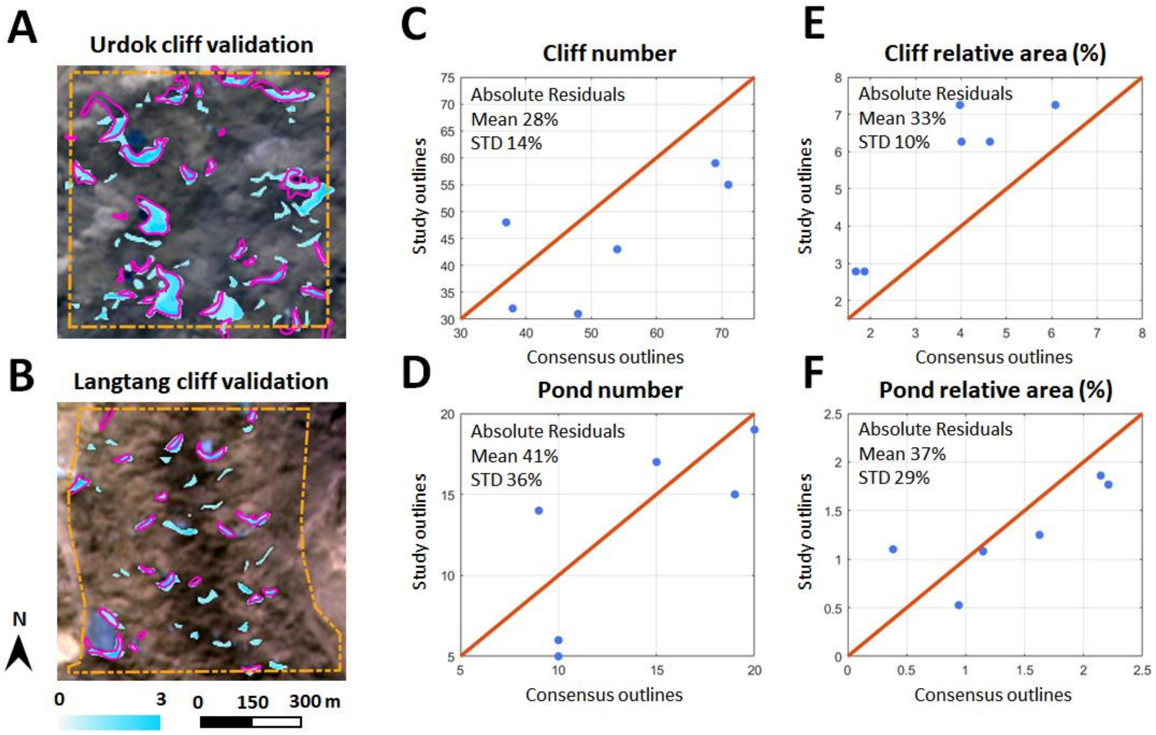

Figure S2: Mapping uncertainties. Panels (A) and (B) show two of the six domains where the cliffs and ponds were validated (orange dashed lines), one on Urdok and the other on Langtang. The pink outlines correspond to the 2011 cliff outlines used in this study and the blue patches are the sum of the non-binary rasterization of outlines from the three independent operators from the same year. The background images are the 2011 RapidEye images (color composites of bands 5, 4 and 2). Panels (C) to (F) represent the cliff and pond relative area and number of this study versus those of the consensus outlines (blue dots), in comparison with the 1:1 line (red). Each dot corresponds to the values from the two domains in each image used for validation.

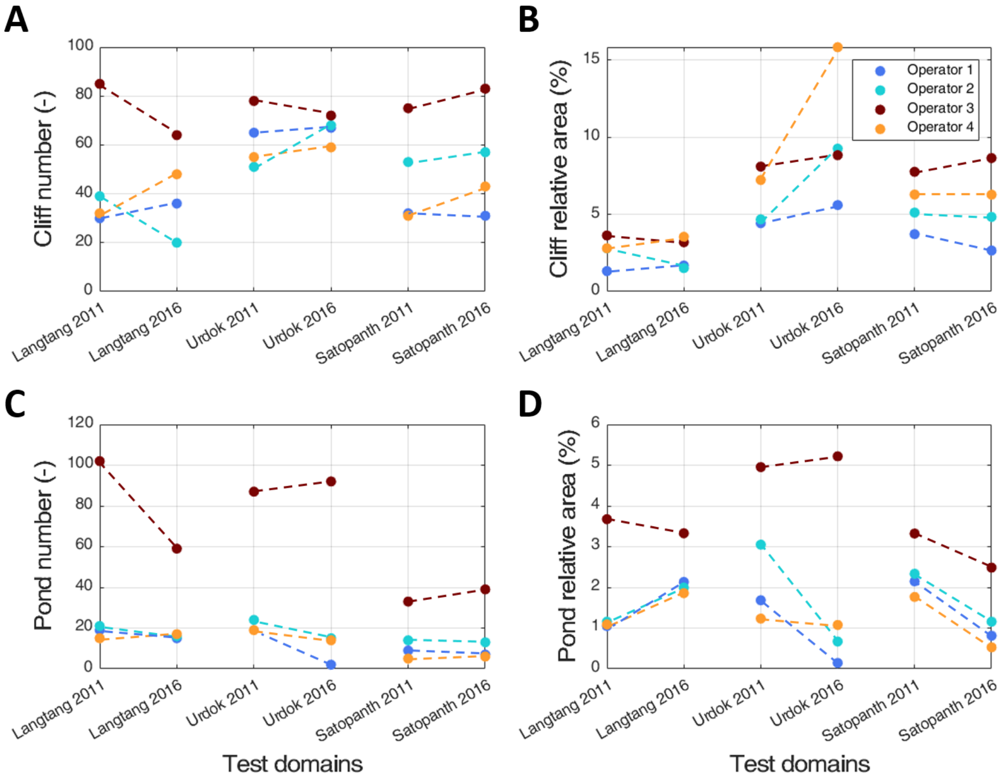

Figure S3: Comparison of the cliffs and ponds number and relative area mapped by the four independent operators in the six validation domains in 2011 and 2016. The different colors correspond to the four operators and the dashed lines represent the trends between 2011 and 2016. Operator 4 was responsible for the cliff and pond delineation in all images outside of the validation period.

Tracking parameters	Values
Maximum distance (between cliffs)	20 $m.yr^{-1}$
Maximum aspect difference	$30°.yr^{-1}$ mod 180 °
Aspect standard deviation threshold over which only the closest pixels are considered	45°

Table S2. Parameters of the tracking algorithm.

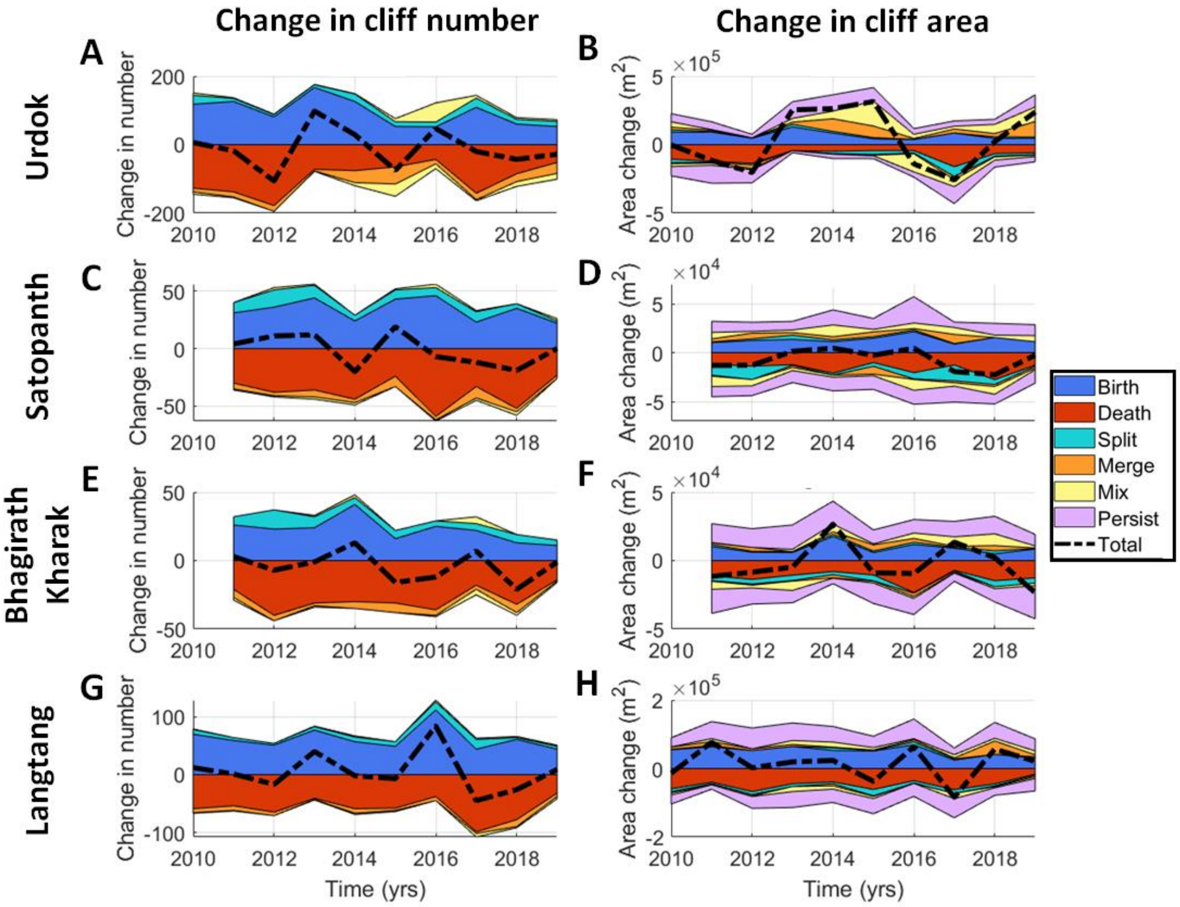

Figure S4: Total contribution of different events to the general evolution of ice cliffs. The left panels show the change in cliff number and the right panels show the change in cliff area. For comparison, the mean cliff number (area) is 328 (6.8×10⁵ m²), 116 (1.5×10⁵ m²), 95 (1.2×10⁵ m²) and 201 (3.3×10⁵ m²) for Urdok, Satopanth, Bhagirath Kharak and Langtang, respectively. Note that the color envelopes are not overlapping but stacked on top of one another.

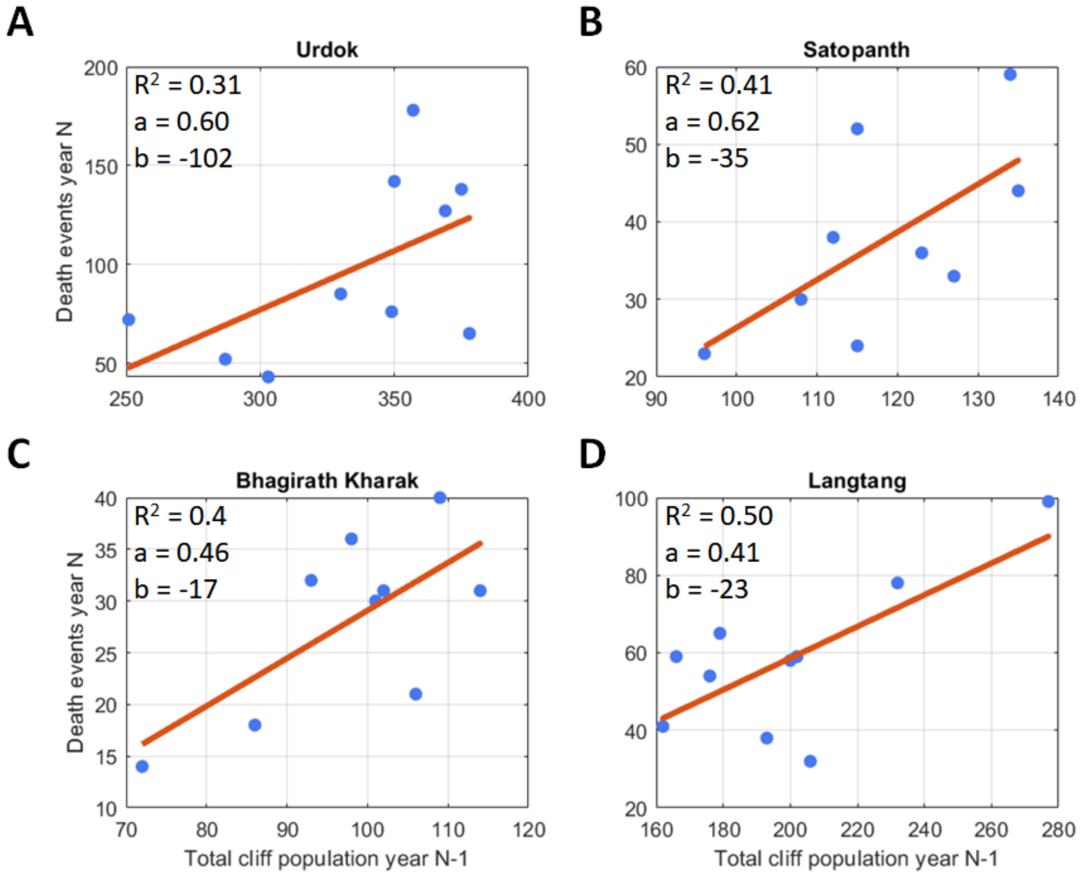

Figure S5: Number of death events plotted against the total number of cliffs the year before at all sites. The red line corresponds to the linear regression between the two datasets of slope a, intercept b, and coefficient of determination R^2.

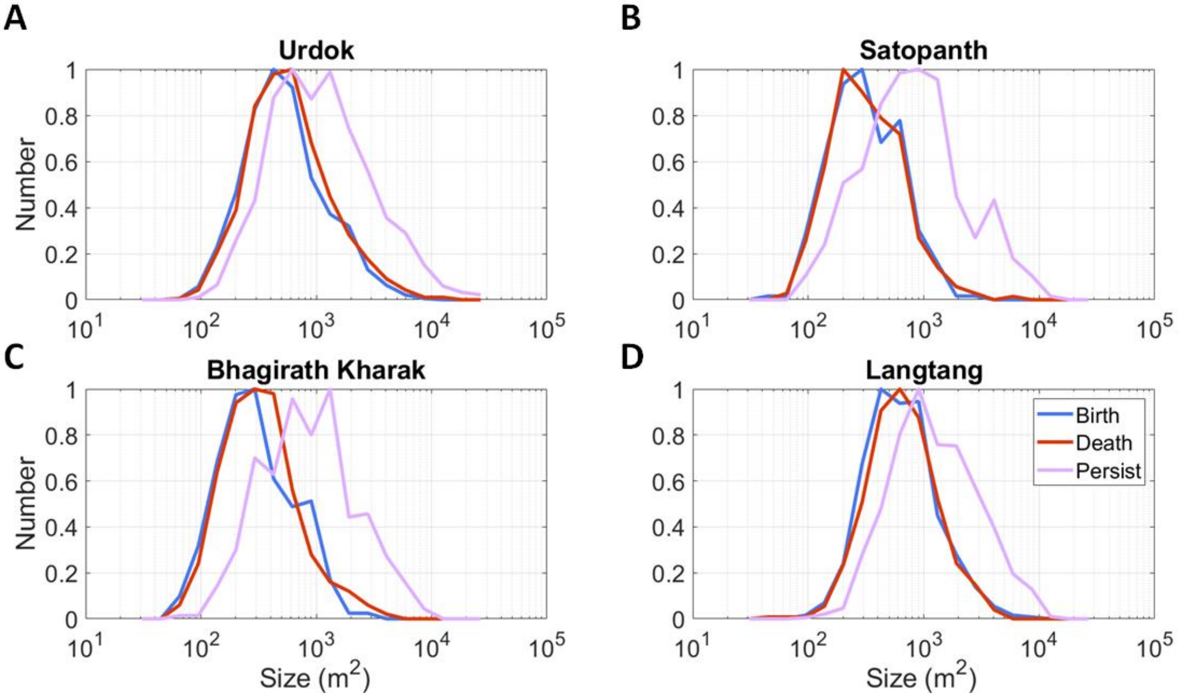

Figure S6: Size distribution of new cliffs, dying cliffs (cliffs gone the next year) and persisting cliffs at each site and calculated over the whole time series.

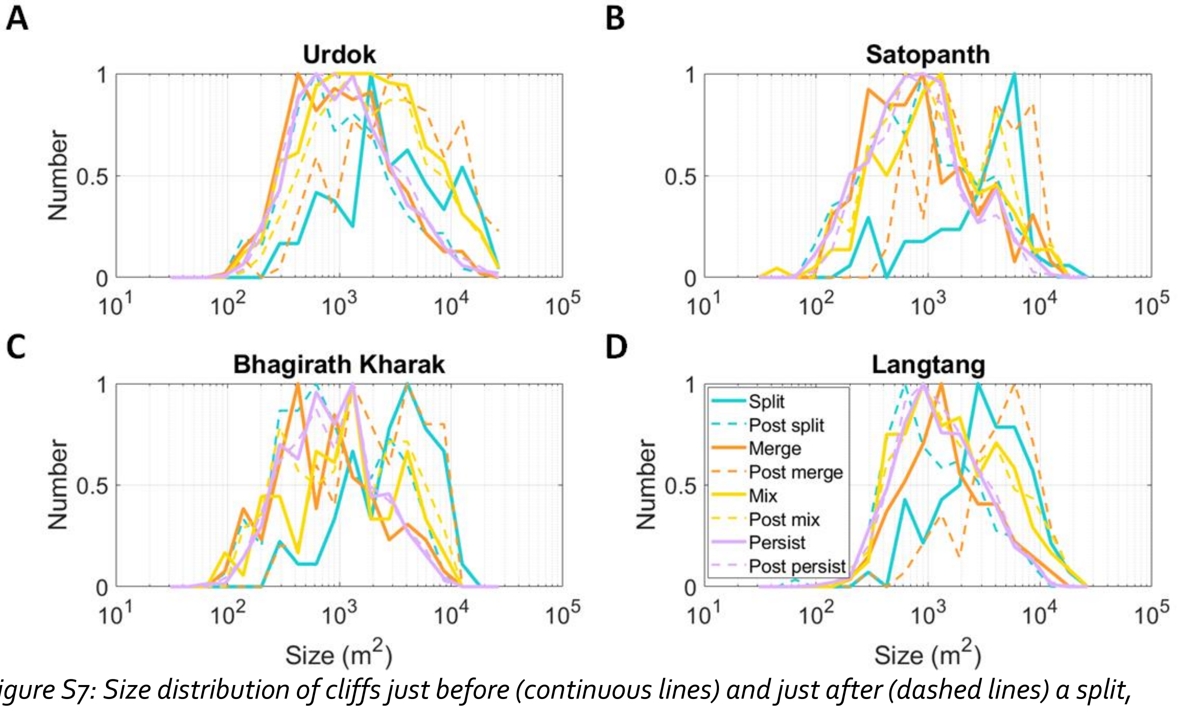

Figure S7: Size distribution of cliffs just before (continuous lines) and just after (dashed lines) a split, merge, mix or persist event at each site and calculated over the whole time series.

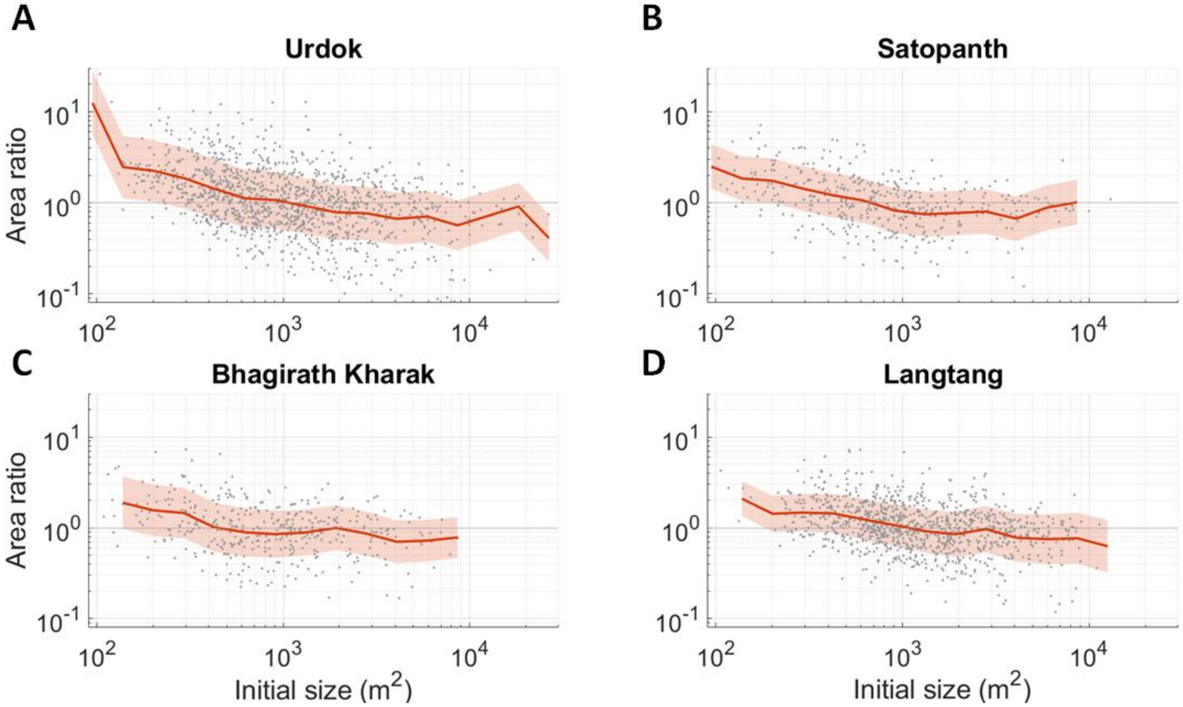

Figure S8: Area ratio of persisting cliffs as a function of the initial size. The grey points correspond to all the persist events of the time series. The data was binned in 19 bins of equal size in the log scale, from 25 m^2 to 32000 m^2. The red lines are the mean area ratio values for these bins and the standard deviation is represented by the light red area.

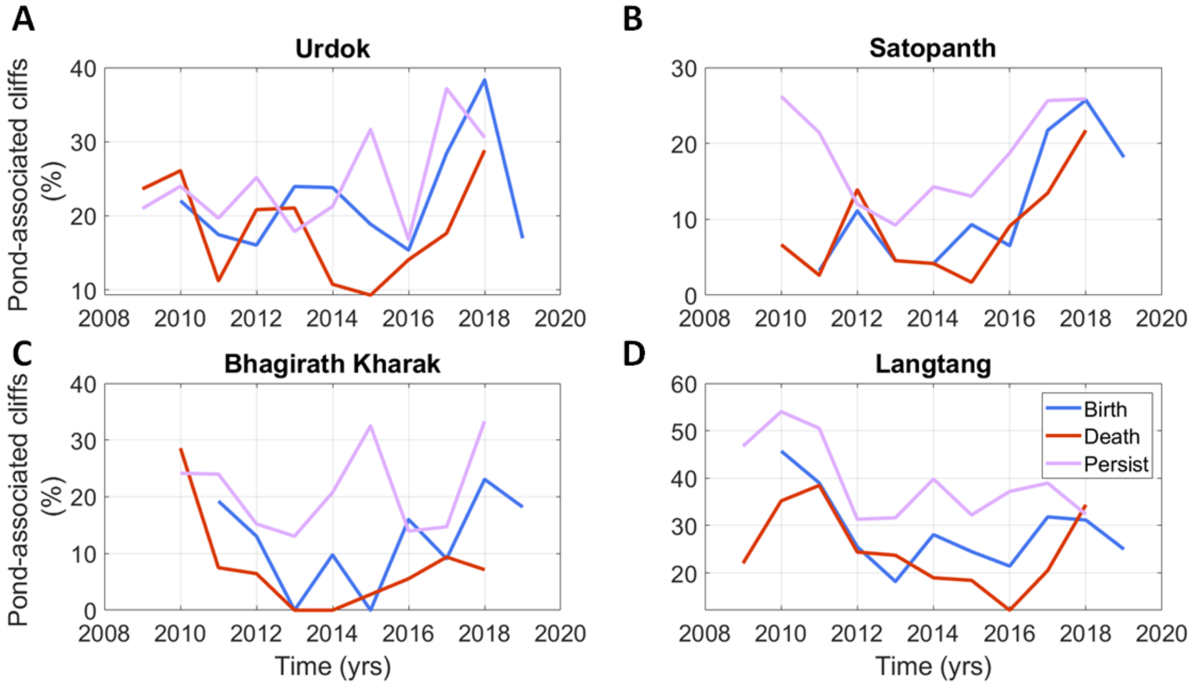

Figure S9: Proportion of cliffs less than 10 m away from a pond in the new cliffs, dying cliffs and persistent cliffs populations, over time and at each site.

Stochastic model description

This stochastic model represents the evolution of each individual cliff of a given population based on different parameters describing the occurrence and characteristics of birth, death, merge, split, mix and persist events (Fig. S10). The model takes as input an initial number of cliffs, each with its own size. At each time step, the cliffs undergo death, split, merge, mix, persist or birth events and their number and sizes change. The only constraint on the cliff sizes calculated from the model is that they are within the minimum and maximum sizes, respectively equal to 100 m^2 (four RapidEye pixels) and the maximum observed cliff size during the period over which parameters are estimated. At each time step, these events are represented following the same procedure (Fig. S10):

1. ***Rates of events***

The number of birth, split, merge and mix events are drawn randomly at each time step from the discrete normal distributions of the yearly number of birth, split, merge and mix events, which are estimated from the data. The number of death events is obtained from the linear regression between death events and cliff population the year before (Eq. 4, Fig. S5) and the stochastic term ξ is given by the normal distribution of the residuals of the linear regression.

2. ***Number of initial cliffs/event***

Death, split, persist and birth events only involve one cliff, but merge and mix events involve two or more cliffs. This number of cliffs per event (initial cliff ratio) is calculated for each merge and mix event based on discrete normal distributions estimated from the data.

3. ***Cliff selection***

The dying cliffs are selected based on the parametrized lognormal distribution of the size of dying cliffs (Fig. S6). For each death event, a number is generated from the lognormal distribution and the cliff with the size closest to this number is selected for this event and removed from the population of cliffs. A similar process is applied to select the cliffs that are to undergo a split or mix event out of the cliffs that have not already been selected to undergo a death event. The cliffs that are to undergo a merge event are randomly selected from the remaining cliffs. All the cliffs that remain undergo a persist event. The size of the new cliffs generated by the birth events are directly generated from the size distribution of new cliffs (Fig. S4).

4. ***Area ratio***

The cliffs undergoing a death event are removed from the population and the size of the new cliffs is generated in the 'Cliff selection' step. For each merge, split, mix and persist event, the final size (sum of final cliff sizes) is calculated from the initial size (sum of initial cliff sizes) and the area ratio (Eq. 1). The area ratio is generated from a lognormal distribution that is dependent on the size of the initial cliffs. The parameters of this size-dependent lognormal distribution, the area ratio mean $\bar{\alpha}$ and variance $Var(\alpha)$ follow a log-linear relationship with the cliff initial sizes (Fig. S6):

(Eq. S1) $$log(\bar{\alpha}) = c_{Mean} \times log(S_i) + d_{Mean}$$

(Eq. S2) $$log(Var(\alpha)) = c_{Var} \times log(S_i) + d_{Var}$$

Where S_i is the cliff initial size, c_{Mean}, c_{Var}, d_{Mean} and d_{Var} are the parameters of the linear regression. The linear regressions are calculated after binning of the data based on their initial size in 19 bins of equal length in the log scale (Fig. S6). In the stochastic model, c_{Mean} and d_{Mean} are fixed, and in a second step, they are expressed as a function of the external drivers based on the results from the multivariate regression.

5. Number of final cliffs/event

Split and mix events result in two or more cliffs. The final size of these events, calculated from the initial size and the area ratio, has to be split between the resulting two or more final cliffs. The number of final cliffs per event (final cliff ratio) is calculated for each individual event from the discrete normal distribution of final cliff ratio. The final size is then randomly distributed between the final cliffs.

This process is repeated for the duration of the modeled period. Since the model is stochastic, each model runs result in a different time series, so the model needs to be run several times to generate a variability range (Fig. S9).

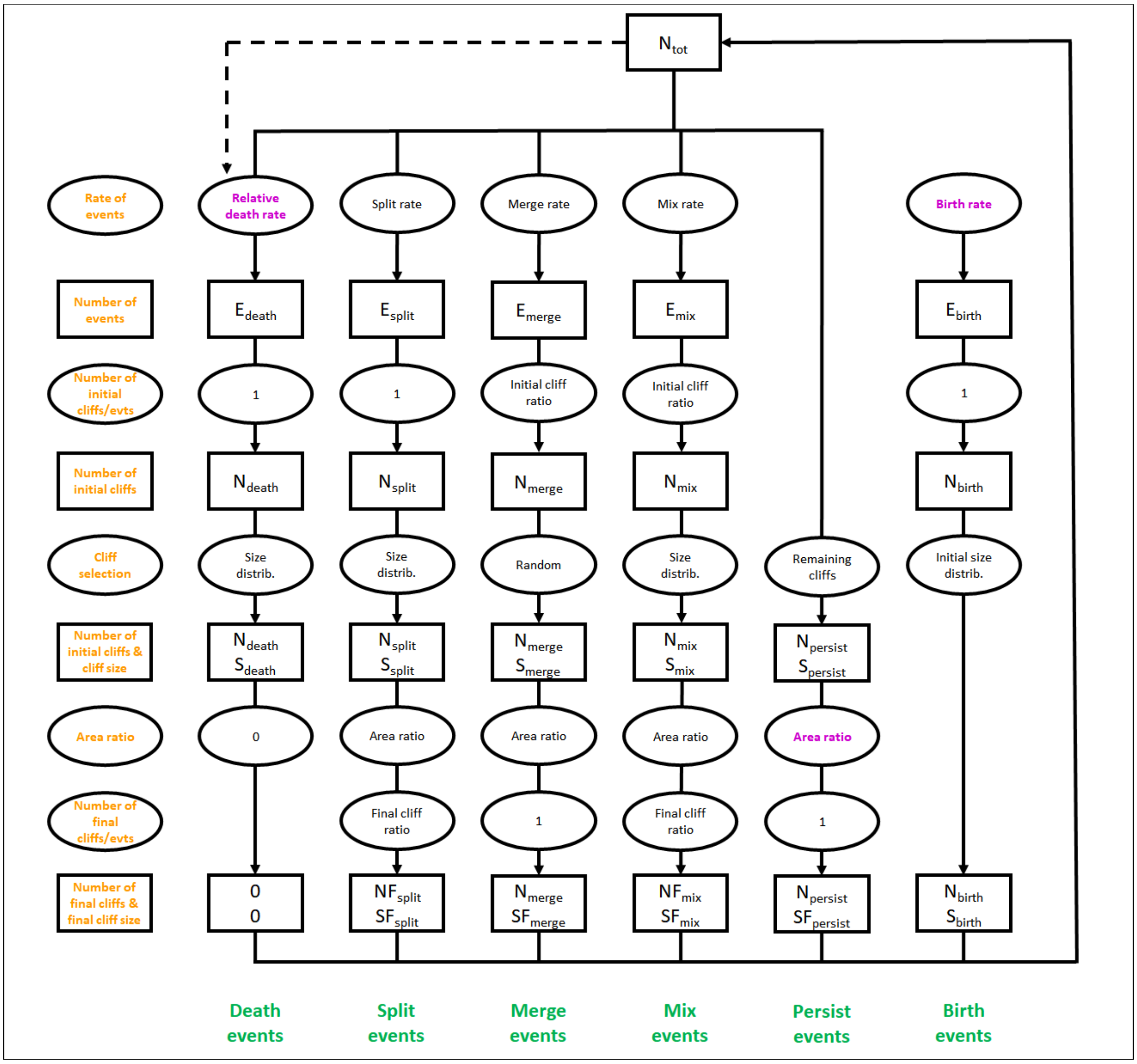

Figure S10: Stochastic birth-death model flowchart. Rectangular boxes describe the state of the system at each step, while the oval boxes describe an action item based on one or more parameters. The orange text describes the general action or state. The purple text corresponds to steps where we add the influence of external drivers in the second version of the model.

Parameters	Probability density function	Model step
	Cliff size limits	
Maximum size	One value (area of largest observed cliff)	All
Minimum size	One value (100 m^2)	All
	Death events	
a	One value	Rate of events
b	One value	Rate of events
ξ	Discrete normal distribution (Mean, Variance)	Rate of events
Size of dying cliff	Lognormal distribution (Mean, Variance)	Cliff selection
	Split events	
Number of split events	Discrete normal distribution (Mean, Variance)	Rate of events
Size of splitting cliff	Lognormal distribution (Mean, Variance)	Cliff selection
Area ratio	Initial size dependent lognormal distribution (Mean, Variance as in Eq. S1 and S2)	Area ratio
Final cliff ratio	Discrete normal distribution (Mean, Variance)	Number of final cliffs/events
	Merge events	
Number of merge events	Discrete normal distribution (Mean, Variance)	Rate of events
Initial cliff ratio	Discrete normal distribution (Mean, Variance)	Number of initial cliffs/event
Area ratio	Initial size dependent lognormal distribution (Mean, Variance as in Eq. S1 and S2)	Area ratio
	Mix events	
Number of mix events	Discrete normal distribution (Mean, Variance)	Rate of events
Initial cliff ratio	Discrete normal distribution (Mean, Variance)	Number of initial cliffs/event
Size of initial cliff	Lognormal distribution (Mean, Variance)	Cliff selection

Area ratio	Initial size dependent lognormal distribution (Mean, Variance as in Eq. S1 and S2)	Area ratio
Final cliff ratio	Discrete normal distribution (Mean, Variance)	Number of final cliffs/events
Persist events		
Area ratio	Initial size dependent lognormal distribution (Mean, Variance as in Eq. S1 and S2)	Area ratio
Birth events		
Number of birth events	Discrete normal distribution (Mean, Variance)	Rate of events
Size of new cliffs	Lognormal distribution (Mean, Variance)	Cliff selection

Table S3. Stochastic model parameters. The parameters were estimated for each glacier over the first five years or the full time series depending on the model runs. a, b and ξ correspond to the parameters in Eq. 3.

Parameters		Values			
		Langtang	Urdok	Satopanth	Bhagirath Kharak
Cliff size limits					
Maximum size (m^2)		8844	21919	13036	7866
Minimum size (m^2)		100	100	100	100
Death events					
a		0.29	0.55	0.49	0.29
b		4	-69	-24	0
ξ	Mean, Variance	-0.02, 0.16	-0.07, 0.33	-0.03, 0.20	-0.04, 0.25
Size of dying cliff	Mean, Variance	6.58, 0.66	6.29, 0.77	5.71, 0.72	5.63, 0.67
Split events					
Number of split events	Mean, Variance	5.6, 1.8	12.4, 7.0	8.0, 3.0	6.0, 1.6
Size of splitting cliff	Mean, Variance	8.3, 0.75	7.5, 0.96	7.8, 1.09	7.8, 0.99
Area ratio	c_{Mean}	-0.71	-0.54	-0.38	-0.38
	d_{Mean}	5.78	4.29	2.81	2.98
	c_{Var}	-0.29	0.12	-0.09	-0.13
	d_{Var}	2.87	-0.27	1.18	1.28
Final cliff ratio	Mean, Variance	2.07, 0.15	2.14, 0.11	2.21, 0.14	2.28, 0.33
Merge events					
Number of merge events	Mean, Variance	5.8, 2.2	14, 8.0	4.6, 2.1	3.8, 1.5
Initial cliff ratio	Mean, Variance	2.06, 0.08	2.14, 0.15	2.13, 0.22	2.25, 0.19

Area ratio	c_{Mean}	-0.08	-0.18	-0.09	-0.21
	d_{Mean}	0.74	1.37	0.90	1.67
	c_{Var}	-0.21	0.29	0.08	-0.01
	d_{Var}	2.26	-1.35	-0.38	0.61
Mix events					
Number of mix events	Mean, Variance	5.2, 2.9	12.4, 6.6	6.6, 1.1	4.4, 1.5
Initial cliff ratio	Mean, Variance	2.36, 0.30	2.43, 0.38	2.33, 0.11	2.38, 0.30
Size of initial cliffs	Mean, Variance	7.5, 0.94	7.0, 1.08	6.9, 1.14	7.0, 1.12
Area ratio	c_{Mean}	0.38	0.44	0.06	-0.14
	d_{Mean}	-3.3	-3.9	-0.4	1.4
	c_{Var}	-0.13	0.05	-0.08	-0.05
	d_{Var}	1.6	0.15	1.08	0.72
Final cliff ratio	Mean, Variance	2.33, 0.33	2.35, 0.21	2.29, 0.26	2.40, 0.49
Persist events					
Area ratio	c_{Mean}	-0.27	-0.30	-0.24	-0.16
	d_{Mean}	2.0	2.2	1.7	1.1
	c_{Var}	0.058	-0.018	-0.031	-0.024
	d_{Var}	0.14	0.85	0.73	0.77
Birth events					
Number of birth events	Mean, Variance	62.8, 10.5	123.6, 30.6	35.6, 8.4	26.0, 9.2
Size of new cliffs	Mean, Variance	6.57, 0.68	6.22, 0.79	5.58, 0.71	5.58, 0.72

Table S4. Stochastic model parameter values for each of the four study glaciers. The parameters are the same as the ones described in Table S3. The parameters shown in this table were estimated for each glacier over the full time series.

Birth rate (num.yr^{-1})	Intercept (num.yr^{-1})	Predictor coefficients								R^2	P-value	RMSE	Adj-R^2
		Air Temp. (°C)	Precip. (mm)	Total pond area (m^2)	Pond area change (m^2.yr^{-1})	Vel. DCG (m.yr^{-1})	Vel. AOI (m.yr^{-1})	Acc. DCG (m.yr^{-2})	Acc. AOI (m.yr^{-2})				
Langtang	-32	64								0.34	0.10	18	0.24
Urdok	416			-4.0e-4		-2.1	-59		27	0.97	0.003	10	0.93
Satopanth	33				-6.8e-4					0.66	0.01	5.6	0.61
Bhagirath Kharak	32			-5.0e-4						0.23	0.23	7.9	0.11

Table S5. Multivariate regression results for the number of birth events. In green are the sites for which the multivariate regression is statistically significant (P value ≤ 0.05). Empty cells show that the corresponding predictors were not included in the multivariate regression of birth rate. The cells colored in orange show that the corresponding predictor was chosen in the first step of the stepwise multivariate regression.

Relative death rate (yr^{-1})	Intercept (yr^{-1})	Predictor coefficients								R^2	P-value	RMSE	Adj-R^2
		Air Temp. (°C)	Precip. (mm)	Total pond area (m^2)	Pond area change (m^2.yr^{-1})	Vel. DCG (m.yr^{-1})	Vel. AOI (m.yr^{-1})	Acc. DCG (m.yr^{-2})	Acc. AOI (m.yr^{-2})				
Langtang	0.31									-	-	0.055	-
Urdok	0.61				2.5e-7	-1.3e-2				0.74	0.02	0.068	0.65
Satopanth	1.92						-0.11	7.6e-2		0.52	0.16	0.070	0.33
Bhagirath Kharak	0.30								-3.1e-2	0.75	0.01	0.036	0.71

Table S6. Multivariate regression results for the relative death rate. In green are the sites for which the multivariate regression is statistically significant (P value ≤ 0.05). Empty cells show that the corresponding predictors were not included in the multivariate regression of relative death rate. The cells colored in orange show that the corresponding predictor was chosen in the first step of the stepwise multivariate regression.

Slope of mean area fraction c_{Mean} (-)	Intercept (-)	Predictor coefficients								R^2	P-value	RMSE	Adj-R^2
		Air Temp. (°C)	Precip. (mm)	Total pond area (m^2)	Pond area change ($m^2.yr^{-1}$)	Vel. DCG ($m.yr^{-1}$)	Vel. AOI ($m.yr^{-1}$)	Acc. DCG ($m.yr^{-2}$)	Acc. AOI ($m.yr^{-2}$)				
Langtang	0.25						-0.08	0.11		0.71	0.03	0.072	0.61
Urdok	0.59	0.13		7.5e-7						0.60	0.07	0.100	0.46
Satopanth	-9.5e-2								-0.11	0.75	0.01	0.050	0.71
Bhagirath Kharak	0.19	-0.10			7.4e-6			-4.0e-2		0.75	0.01	0.036	0.71

Table S7. Multivariate regression results for the mean area fraction slope of the persist events. In green are the sites for which the multivariate regression is statistically significant (P value ≤ 0.05). Empty cells show that the corresponding predictors were not included in the multivariate regression of the mean area fraction slope. The cells colored in orange show that the corresponding predictor was chosen in the first step of the stepwise multivariate regression.

Intercept of mean area fraction d_{Mean} (-)	Intercept (-)	Predictor coefficients								R^2	P-value	RMSE	Adj-R^2
		Air Temp. (°C)	Precip. (mm)	Total pond area (m^2)	Pond area change ($m^2.yr^{-1}$)	Vel. DCG ($m.yr^{-1}$)	Vel. AOI ($m.yr^{-1}$)	Acc. DCG ($m.yr^{-2}$)	Acc. AOI ($m.yr^{-2}$)				
Langtang	-1.6						0.55	-0.78		0.70	0.03	0.51	0.60
Urdok	-4.7	-1.0		-4.9e-6						0.60	0.07	0.73	0.46
Satopanth	0.68								0.76	0.75	0.01	0.37	0.70
Bhagirath Kharak	-1.8	0.82						0.33		0.71	0.05	0.53	0.59

Table S8. Multivariate regression results for the mean area fraction intercept of the persist events. In green are the sites for which the multivariate regression is statistically significant (P value ≤ 0.05). Empty cells show that the corresponding predictors were not included in the multivariate regression of the mean area fraction intercept. The cells colored in orange show that the corresponding predictor was chosen in the first step of the stepwise multivariate regression.

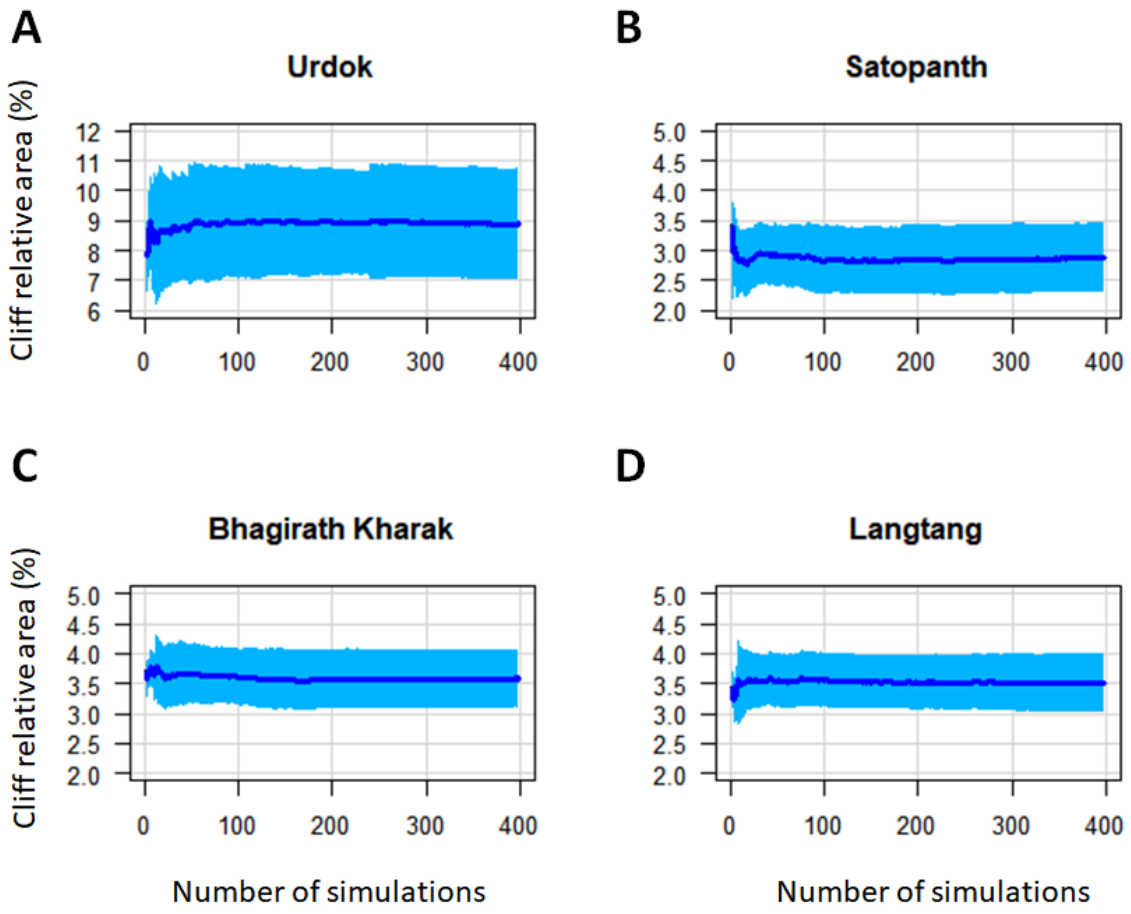

Figure S11: Mean (blue line) and standard deviation (mid blue area) of the modeled cliff relative area after 10 years as a function of the number of simulations.

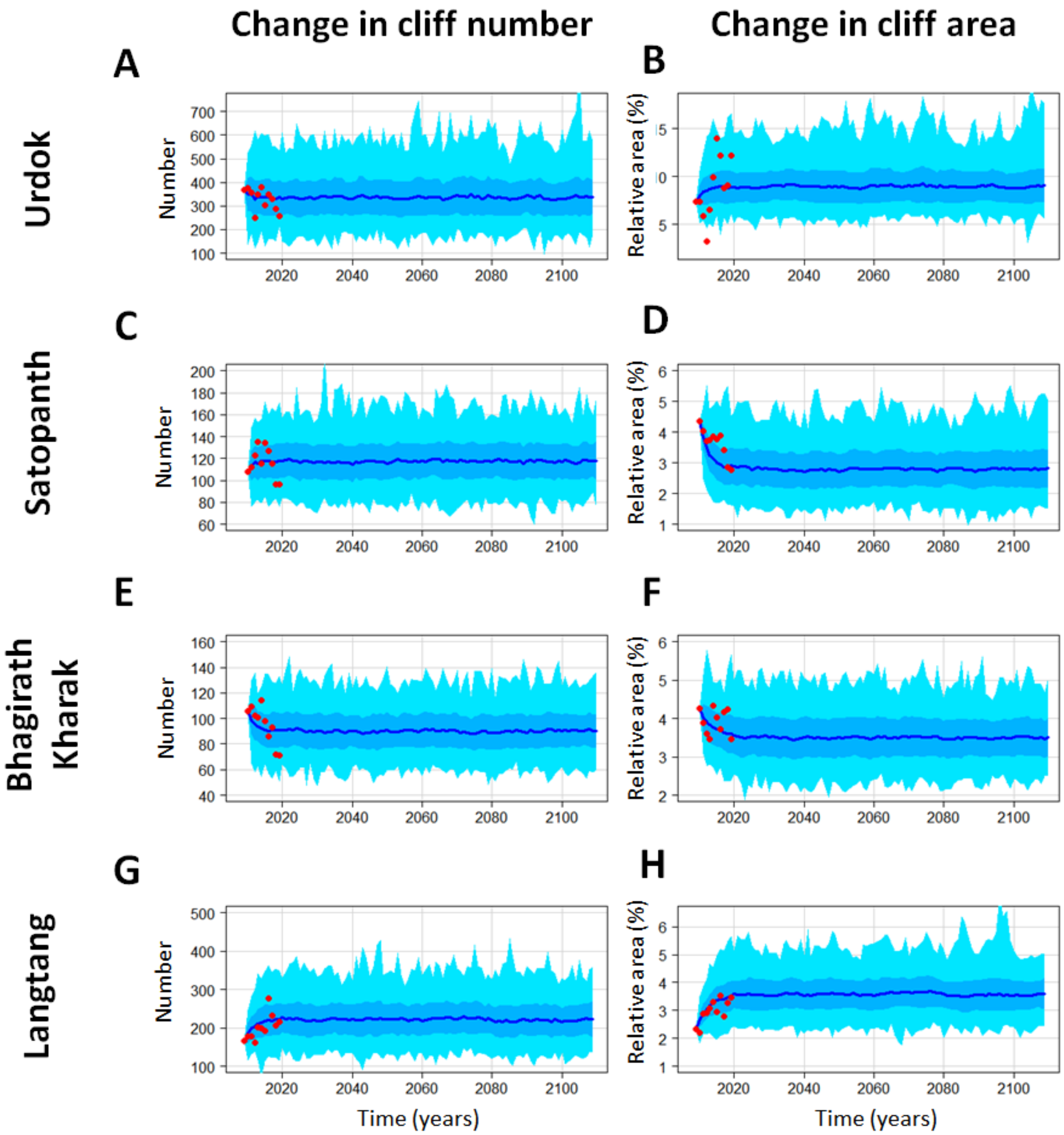

Figure S12: Outputs from the stochastic model for the number of cliffs and their relative area. The model was run 200 times from 2009 (2010 for Satopanth and Bhagirath Kharak) to 2120. Parameters were estimated over the full time series. The red dots correspond to the observations from the mapping. The dark blue line corresponds to the average values of the 200 simulation runs. The mid blue area represents the standard deviation of the runs and the light blue area the maximum and minimum values.

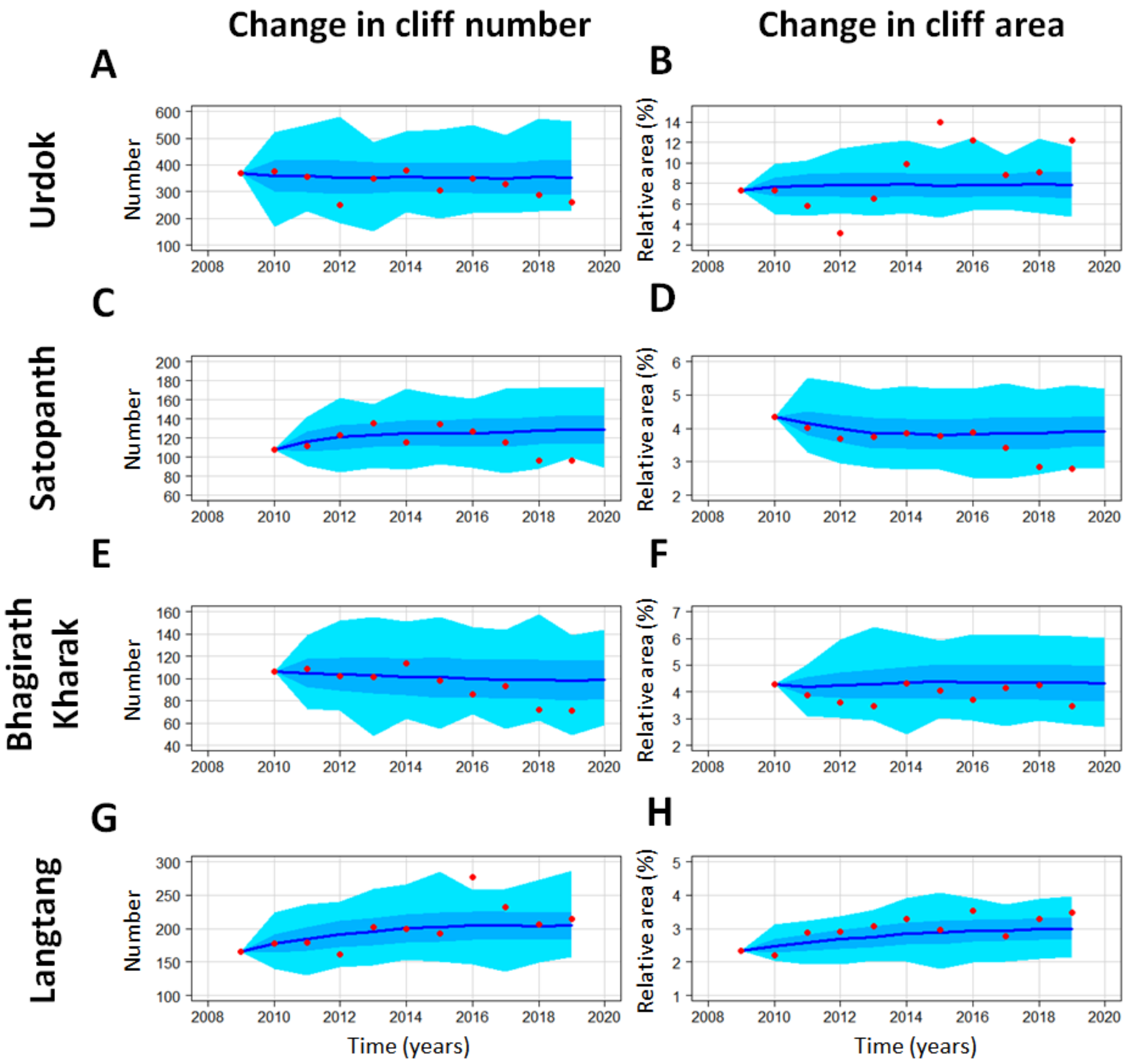

Figure S13: Outputs from the stochastic model for the number of cliffs and their relative area. The model was run 200 times from 2009 (2010 for Satopanth and Bhagirath Kharak) to 2019. Parameters were estimated over the first 5 years of the time series. The red dots correspond to the observations from the mapping. The dark blue line corresponds to the average values of the 200 simulation runs. The mid blue area represents the standard deviation of the runs and the light blue area the maximum and minimum values.

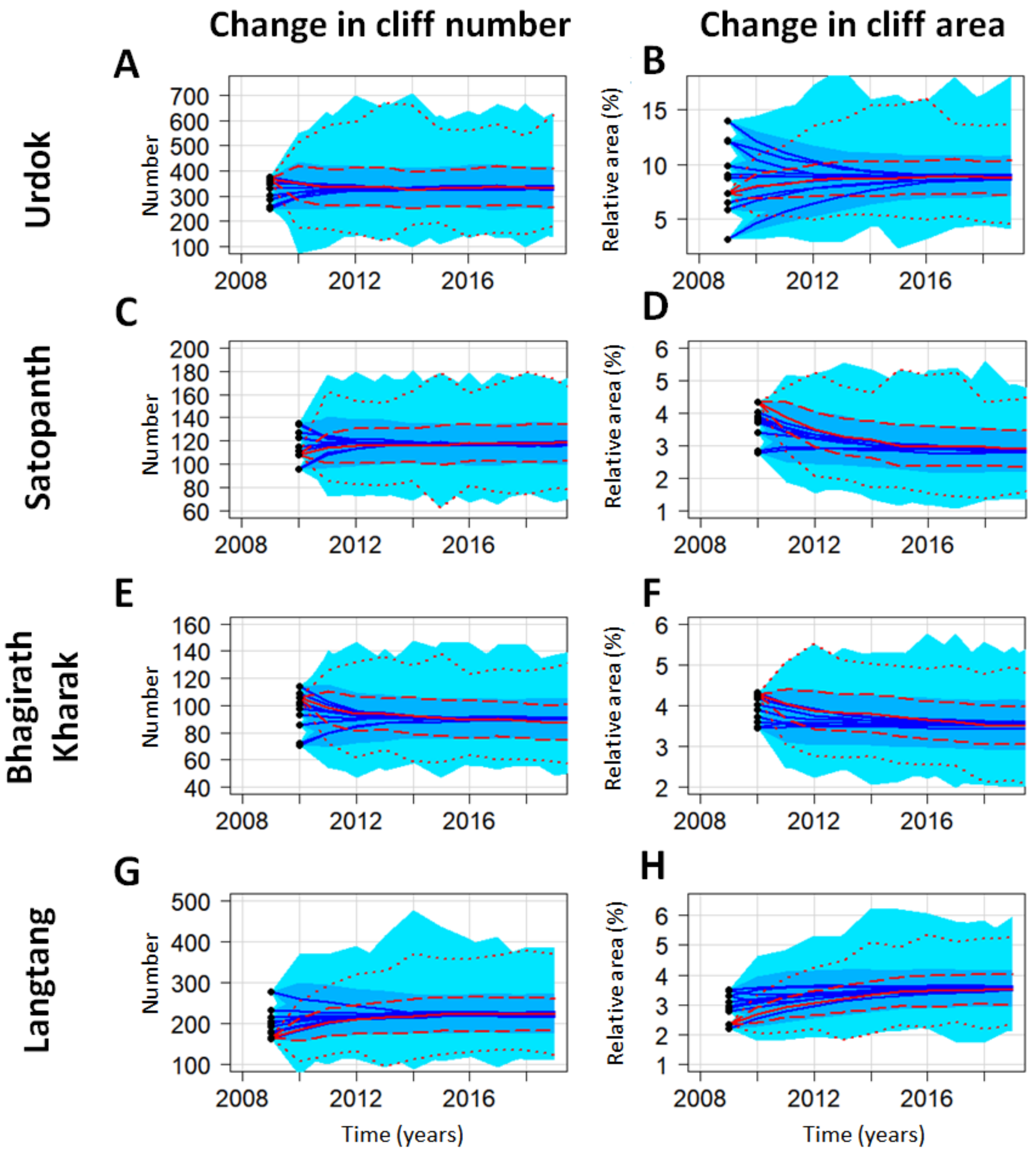

Figure S14: Outputs from the stochastic model for the number of cliffs and their relative area for different initial conditions (black dots), corresponding to each year of the time series. The model was run 200 times from 2009 (2010 for Satopanth and Bhagirath Kharak) to 2019 for each initial condition. Parameters were estimated over the first 10 years of the time series. The dark blue lines correspond to the average values of the 200 simulation runs. The mid blue areas represent the standard deviation of the runs and the light blue areas the maximum and minimum values. The red lines correspond to the average (solid), standard deviation (long dashes) and extreme extents (dots) for the 2009 initial conditions
